# Adverse drug events associated with linezolid administration: a real-world pharmacovigilance study from 2004 to 2023 using the FAERS database

**DOI:** 10.3389/fphar.2024.1338902

**Published:** 2024-02-16

**Authors:** Fan Zou, Zhiwei Cui, Siyu Lou, Yingyong Ou, Chengyu Zhu, Chengjie Shu, Junyou Chen, Ruizhen Zhao, Zhu Wu, Li Wang, Zhenyong Chen, Huayu Chen, Yuanbo Lan

**Affiliations:** ^1^ Department of Tuberculosis, Affiliated Hospital of Zunyi Medical University, Zunyi, Guizhou, China; ^2^ Department of Obstetrics and Gynecology, The First Affiliated Hospital of Xi’an Jiaotong University, Xi’an, China; ^3^ Department of Respiratory and Critical Care Medicine, Affiliated Hospital of Zunyi Medical University, Zunyi, China

**Keywords:** linezolid, adverse drug event, FAERS, disproportionality analysis, pharmacovigilance, real-world analysis

## Abstract

**Introduction:** Linezolid is an oxazolidinone antibiotic that is active against drug-resistant Gram-positive bacteria and multidrug-resistant *Mycobacterium tuberculosis*. Real-world studies on the safety of linezolid in large populations are lacking. This study aimed to determine the adverse events associated with linezolid in real-world settings by analyzing data from the US Food and Drug Administration (FDA) Adverse Event Reporting System (FAERS).

**Methods:** We retrospectively extracted reports on adverse drug events (ADEs) from the FAERS database from the first quarter of 2004 to that of 2023. By using disproportionality analysis including reporting odds ratio (ROR), proportional reporting ratio (PRR), Bayesian Confidence Propagation Neural Network (BCPNN), along with the multi-item gamma Poisson shrinker (MGPS), we evaluated whether there was a significant association between linezolid and ADE. The time to onset of ADE was further analyzed in the general population and within each age, weight, reporting population, and weight subgroups.

**Results:** A total of 11,176 reports of linezolid as the “primary suspected” drug and 263 significant adverse events of linezolid were identified, including some common adverse events such as thrombocytopenia (*n* = 1,139, ROR 21.98), anaemia (*n* = 704, ROR 7.39), and unexpected signals that were not listed on the drug label such as rhabdomyolysis (*n* = 90, ROR 4.33), and electrocardiogram QT prolonged (*n* = 73, ROR 4.07). Linezolid-induced adverse reactions involved 27 System Organ Class (SOC). Gender differences existed in ADE signals related to linezolid. The median onset time of all ADEs was 6 days, and most ADEs (*n* = 3,778) occurred within the first month of linezolid use but some may continue to occur even after a year of treatment (*n* = 46).

**Conclusion:** This study reports the time to onset of adverse effects in detail at the levels of SOC and specific preferred term (PT). The results of our study provide valuable insights for optimizing the use of linezolid and reducing potential side effects, expected to facilitate the safe use of linezolid in clinical settings.

## 1 Introduction

Linezolid, an oxazolidinone antibiotic, is effective against drug-resistant Gram-positive bacteria such as methicillin-resistant *Staphylococcus aureus*, vancomycin-resistant *Enterococcus*, and multidrug-resistant *Mycobacterium tuberculosis* (Sotgiu et al., 2012; Hashemian et al., 2018; Crass et al., 2019). It targets the bacterial ribosomes and inhibits protein synthesis, thereby preventing the formation of the initiation complex (Ippolito et al., 2008; Wilson et al., 2008; Long et al., 2010; Long and Vester, 2012). Linezolid reduces toxin production by Gram-positive pathogens (Ippolito et al., 2008). Owing to its high bioavailability, it can be administered intravenously or orally without dosage adjustment (Welshman et al., 2001). Linezolid is considered a first-line antibiotic for methicillin-resistant *S. aureus* pneumonia and is more cost-effective and reduces mortality significantly compared to vancomycin in the treatment of methicillin-resistant *S. aureus* (MRSA) infections (Liu et al., 2011; Niederman et al., 2014; Collins and Schwemm, 2015; von Dach et al., 2017). Linezolid has been approved for the treatment of hospital-acquired pneumonia caused by *S. aureus*, vancomycin-resistant *Enterococcus faecalis* (VREF) infections, complicated skin and skin structure infections (SSSIs), uncomplicated SSSIs caused by meticillin-sensitive S.aureus (MSSA) or *Streptococcus* pyogenes, community-acquired pneumonia, and pneumococcal meningitis caused by penicillin-resistant *Streptococcus* pneumoniae. Linezolid is associated with lower mortality rates compared to daptomycin in the treatment of vancomycin-resistant *Enterococcus* (VRE) bacteremia, with lower infection-related and hospitalization mortality rates. Moreover, linezolid is effective in treating multidrug-resistant tuberculosis (TB) (Cox and Ford, 2012; Brown et al., 2015; Hughes et al., 2015; Tang et al., 2015). Overall, linezolid exerts promising therapeutic effects and has been approved by the U.S. FDA for treating various infections.

In Phase III and IV clinical studies and randomized controlled trials, the most common adverse drug reactions (ADRs) of linezolid use included gastrointestinal reactions, including diarrhea, nausea and vomiting, bone marrow suppression, peripheral neuropathy, and headache (Rubinstein et al., 2001; Prokocimer et al., 2013; O'Riordan et al., 2019; Esmail et al., 2022; Kotsaki et al., 2023). Fortunately, most ADRs did not result in serious adverse outcomes and given the strict diagnostic criteria, selection criteria, relatively small sample size, and limited follow-up time, ADRs targeted single or limited number of systems. Linezolid has been approved for extensively drug-resistant TB and multidrug-resistant TB, which may result in the development of some ADR exacerbations or previously unidentified safety concerns. Data on the combined safety profile of linezolid from large samples and in the real world are lacking. With the expansion of indications for linezolid, it is now being widely used in clinical settings. Therefore, post-marketing evaluation of linezolid using data mining is necessary.

The FDA Adverse Event Reporting System (FAERS) database is a valuable resource for post-marketing surveillance and early detection of drug safety issues (Cirmi et al., 2020; Feng et al., 2022). It contains real adverse event reports from various sources, including those documented by healthcare professionals, consumers, and manufacturers. The database is regularly updated and publicly available for download on the FDA website (Sakaeda et al., 2013). Considering the lack of evidence of adverse events of linezolid at the real-world level, we conducted a post-marketing surveillance to assess adverse events associated with linezolid use in FAERS from the first quarter of 2004 to that of 2023. We comprehensively analyzed system-specific side effects of linezolid and their time of onset as well as gender-based differences. The results of this study can guide physicians and health policymakers in monitoring adverse drug reactions and providing recommendations for the safe clinical use of linezolid.

## 2 Materials and methods

### 2.1 Data sources and preprocessing

**FIGURE 1:**
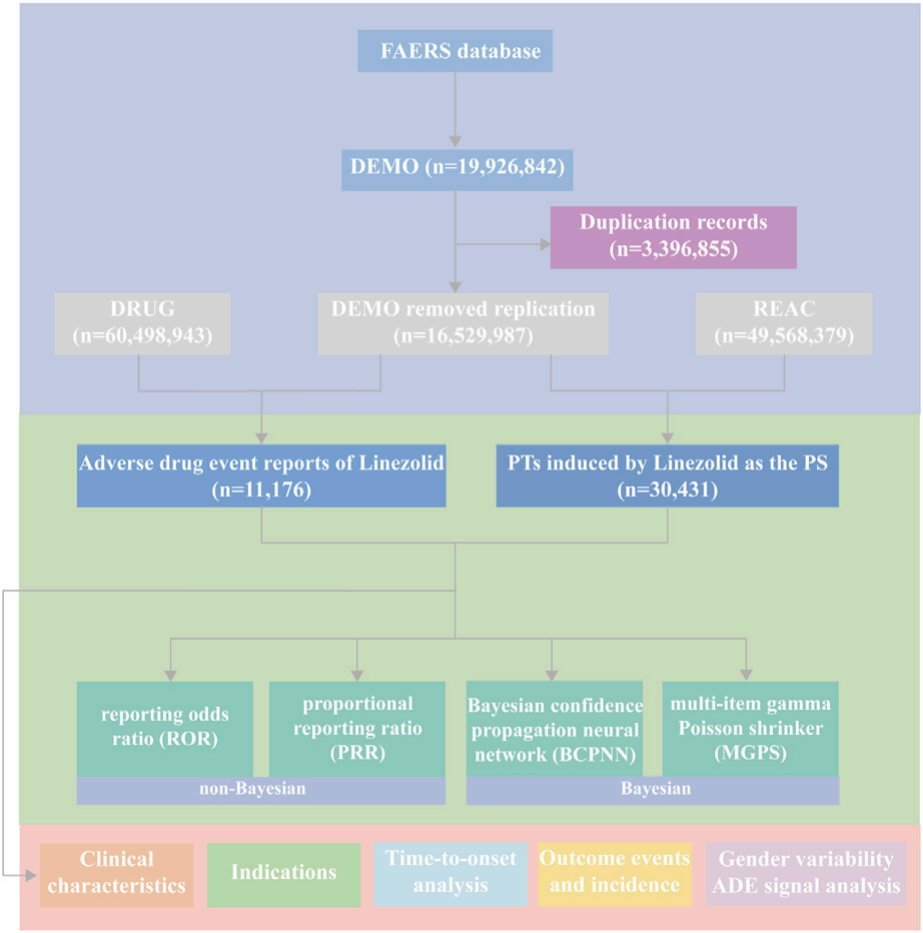
A flowchart of the whole study.

We conducted this retrospective pharmacovigilance analysis using the FAERS database. FAERS is a compilation of adverse drug event (ADE) reports and allows researchers to perform signal detection and quantify the associations between drug dosing and ADEs (Sakaeda et al., 2013). The FAERS database is updated quarterly and comprises seven datasets on demographic and administrative information (DEMO), drug information (DRUG), adverse drug reaction information (REAC), patient outcomes information (OUCT), reported sources (RPSR), drug therapy start dates and end dates (THER), and indications for drug administration (INDI). There are unavoidable cases of duplicate reporting in FAERS due to the characteristics of data updating. Therefore, we set the retrieval timeframe from 1 January 2004, to 31 March 2023, and removed duplicates to enhance the reliability of the findings based on the following criteria recommended by the FDA (Shu et al., 2022; Wang et al., 2023): if CASEIDs were the same, the most recent FDA_DT was chosen; if CASEIDs and FDA_DTs were the same, the higher PRIMARYID was chosen (Cui et al., 2023). After data preprocessing, we obtained 16,529,987 DEMO reports, 60,498,943 DRUG cases, and 49,568,379 REAC records (Figure 1).

### 2.2 Study design (drug selection and signal detection)

Because FAERS does not utilize a uniform drug coding system, both generic (LINEZOLID) and brand (ZYVOXID) names were used to identify ADEs associated with linezolid (see Supplementary Table S1 for the detailed list of drug names used for search). Drugs reported in FAERS were categorized into four patterns, namely PS (primary suspect), SS (second suspect), C (concomitant), and I (interacting). To improve accuracy, only drugs with linezolid as the PS was retained in the role codes for ADEs, resulting in 11,176 ADE reports (linezolid-related ADEs) screened for linezolid administration (Ji et al., 2022a). Medical Dictionary for Regulatory Activities (MedDRA) is a standardized medical terminology that globally facilitates the recording and reporting of ADE data (Brown, 2004; Singh, 2015). Its hierarchical structure encompasses multiple levels, ranging from lower terminology to system organ class (SOC) (Brown, 2003). SOC is the highest level of terminology in MedDRA used for classifying and reporting adverse events in the Drug Safety Monitoring and Reporting System. To summarize and analyze ADE features in a structured way, all ADEs in our collection were coded using the preferred term (PT) and then mapped to their corresponding highest SOC level in MedDRA (version 26.0) (Wang et al., 2014; Zhou et al., 2023). In total, 30,431 PTs induced by linezolid as the PS (linezolid-related PTs) were identified.

In pharmacovigilance studies, disproportionality analysis is an instrumental method for identifying and detecting drug-related adverse reaction signals (van Puijenbroek et al., 2002). To improve the results’ reliability, we employed different methods of disproportionality analysis, including two non-Bayesian methods(the reporting odds ratio [ROR] and the proportional reporting ratio [PRR]) and two Bayesian methods, including the Bayesian Confidence Propagation Neural Network (BCPNN), along with the multi-item gamma Poisson shrinker (MGPS) (Trippe et al., 2017). Non-Bayesian methods such as ROR may exhibit better efficacy for early signal detection, while the Bayesian approach has a strong detection power for unique signals even when there are few ADEs reported for the drug (Chen et al., 2008; Nomura et al., 2015). The two-by-two contingency table and detailed formulas for these methods of disproportionality analysis and the positive signal thresholds are provided in Table 1. Beyond the threshold, a larger value indicates a stronger signal value. We indicated signals not listed in the drug label as “unexpected signals.” To enhance the reliability of the findings, separate disproportionality analyses were performed based on patient age, gender, weight, and reporting sources.

**TABLE 1:** A two-by-two contingency table and detailed formulas for disproportionality analysis.

	Target adverse drug event	Other adverse drug events	Sums
Linezolid	a	b	a+b
Other drugs	c	d	c+d
Sums	a+c	b+d	a+b+c+d

Algorithms	Equation	Criteria
R	ROR=ad/bc	lower limit of 95% CI>1, N≥3
	$95\%\text{CI}=e\ln\left(\text{ROR}\right)\pm 1.96(1/a+1/b+1/c+1/d\hat{)}0.5$	
PRR	$\text{PRR}=\left[a\left(c+d\right)\right]/\left[c\left(a+b\right)\right]$	PRR≥2, χ2 ≥4, N≥3
BCPNN	$\chi 2=\left[\left(\text{ad}-\text{bc}\hat{)}2\right.\right]\left(a+b+c+d\right)/\left[\left(a+b\right)\left(c+d\right)\left(a+c\right)\left(b+d\right)\right]\text{IC}=\log2a\left(a+b+c+d\right)/\left[\left(a+c\right)\left(a+b\right)\right]$	IC025>0
	95%CI=EIC ± 2VIC^0.5	
MGPS	$\text{EBGM}=a\left(a+b+c+d\right)/\left[\left(a+c\right)\left(a+b\right)\right]$	EBGM05>2
	$95\%\text{CI}=e\ln\left(\text{EBGM}\right)\pm 1.96(1/a+1/b+1/c+1/d\hat{)}0.5$	

The formulas to calculate the signal strength are as follows: a, number of reports containing both the target drug and the target adverse drug event; b, number of reports containing other adverse drug events of the target drug; c, number of reports containing the target adverse drug event of other drugs; d, number of reports containing other drugs and other adverse drug events. Abbreviations: 95% CI, 95% confidence interval; N, the number of reports; χ2, chi-squared; ROR, reporting odds ratio; PRR, proportional reporting ratio; BCPNN, Bayesian confidence propagation neutral network; MGPS, multi-item gamma Poisson shrinker; IC, information component; IC025, the lower limit of the 95% CI of the IC; E (IC), the IC expectations; V (IC), the variance of IC; EBGM, empirical Bayesian geometric mean; EBGM05, the lower limit of the 95% CI of EBGM.

Additionally, to discern the disproportional signals between male and female following linezolid administration, we employed the formula of ROR method. The ROR used here does not strictly adhere to the pharmacoepidemiological definition of ROR, as elucidated in the caption of Supplementary Table S4. According to the 2 by 2 contingency table, we calculated the *p*-value based on the chi-square (χ2) test. We generated a volcano plot displaying the log2-transformed ROR values on the horizontal axis and the -log10-transformed corrected *p*-values (*P*.adj, adjusted by FDR) on the vertical axis, utilizing the R package “ggplot2” (version 3.3.6) (Li et al., 2023). When the ROR is greater than 1 and the *P*.adj is greater than 0.05, it suggests that female patients are more likely than male patients to report a specific ADE. Conversely, when the ROR is less than 1 and the *P*.adj is less than 0.05, it suggests that male patients are more likely than female patients to report a specific ADE.

### 2.3 Time to onset (TTO) analysis

The TTO of linezolid-related ADEs is defined as the time interval between the ADE onset date in the DEMO file (EVENT_DT) and the date of medication initiation in the THER file (START_DT). Inaccurate or missing dates, and cases with ADE onset dates earlier than the start date of linezolid medication were excluded. The frequency of adverse events post-therapy initiation is contingent upon the drug’s mechanism of action and may fluctuate over time. In contrast, adverse events unrelated to drug therapy transpire at a consistent rate (Cornelius et al., 2012). The Weibull distribution test determines the proportional change in the adverse event rate, indicating the risk of increase or decrease over time. Consequently, we conducted a comprehensive TTO assessment based on median, quartile, extremes, and the Weibull distribution test (Kinoshita et al., 2020). The Weibull distribution curve is defined by two primary parameters: the scale parameter (α) and the shape parameter (β). For the purposes of this study, only parameter β is considered and discussed. If the shape parameter β <1 and its 95% confidence interval (CI) <1, the risk of adverse reactions is considered to decrease over time (early failure type curve); if the shape parameter β is approximately equal to or close to 1 and its 95% CI contains the value of 1, it is estimated that the risk occurs constantly over time (random failure type curve); and if the shape parameter β >1 and its 95% CI excludes the value of 1, the hazard is considered to increase over time (wear failure type curve) (Sauzet et al., 2013; Mazhar et al., 2021). In order to enhance the reliability of the analyses, Weibull distribution test was performed in the overall and subgroups respectively.

### 2.4 Statistical analysis

**FIGURE 2:**
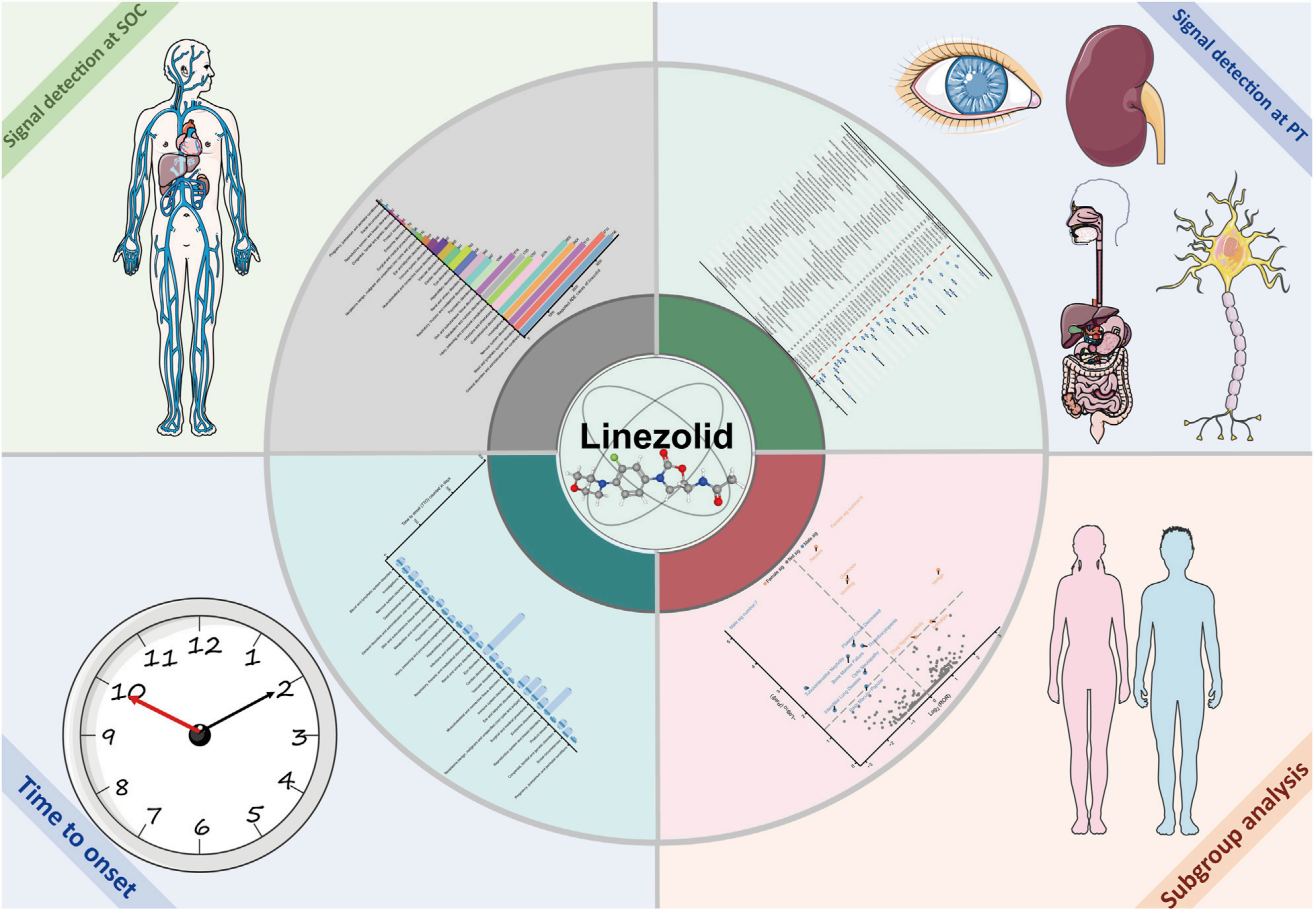
The main findings of this study. After data cleaning, we detected the signal strength of ADEs at the SOC and PT levels. Moreover, we performed gender-based subgroup analysis and conducted detailed calculations and comparisons of TTO. ADEs, adverse drug events; SOC, System Organ Class; PT, preferred term; TTO, time to onset.

Differences in values among multiple groups were assessed using the Kruskal–Wallis test and Dunn’s test. SAS 9.4 and Microsoft EXCEL 2019 were used to process the data. The R (version 4.2.1) language was used for data visualization and statistical calculations. The 3D structure of linezolid was obtained from PubChem (https://pubchem.ncbi.nlm.nih.gov/) (Kim et al., 2023). The image source in Figure 2 is Servier Medical Art (https://smart.servier.com/), provided by Servier, licensed under a Creative Commons Attribution 3.0 unported license.

## 3 Results

### 3.1 Descriptive characteristics

In this study, 16,529,987 reported cases were collected from the FAERS database during the study period (Q1 2004–Q1 2023), and 11,176 linezolid-related ADEs and 30,431 linezolid-related PTs were finally obtained after removing duplicates. The demographic characteristics of linezolid-associated ADEs are described in Table 2. The number of reports identifying the gender of the submitters was 9,250, of which 5,222 were submitted by male (46.73%) and 4,028 by female (36.04%). The number of reports containing age-specific information was 319, with 19 (0.17%), 162 (1.45%), and 138 (1.23%) reports for <18, 18–64, and >64 years of age, respectively. Weight data were available for 3,372 patients, with the group<80 kg accounting for the largest proportion (20.20%).

**TABLE 2:** Demographic characteristics of ADEs reported in the FAERS database (January 2004-March 2023) with linezolid as the primary suspect drug.

Characteristics	Case number	Case proportion, %
Gender, n (%)		
F	4,028	36.04%
M	5,222	46.73%
Unknown	1,926	17.23%
Age		
<18	19	0.17%
18–64	162	1.45%
>64	138	1.23%
Unknown	10,857	97.15%
Weight		
<80	2,258	20.20%
80–100	701	6.27%
>100	413	3.70%
Unknown	7,804	69.83%
Reported Countries (top five)		
US	2,683	24.01%
FR	1,204	10.77%
JP	1,089	9.74%
GB	985	8.81%
CN	645	5.77%
Reported person		
Health professionals	9,472	84.76%
Consumer	1,261	11.28%
Unknown	443	3.96%
Outcome		
HO	3,595	27.92%
LT	934	7.26%
DS	268	2.09%
RI	98	0.76%
DE	1,440	11.20%
OT	6,441	50.07%
Unknown	90	0.70%
Indication (top five)		
Staphylococcal infection	1,081	9.67%
Tuberculosis	673	6.02%
Infection	552	4.94%
Pneumonia	396	3.54%
Enterococcal infection	355	3.18%

F, female; M, male; US, United States; FR, France; JP, Japan; GB, Great Britain; CN, China; HO, hospitalization; LT, life-threatening; DS, disability; RI, required intervention; DE, death; OT, other serious outcomes; ADEs, adverse drug events.

The country with the most documented information was United States (24.10%), followed by France (10.77%), Japan (9.74%), Great Britain (8.81%), and China (5.77%). The majority of reports submitted were by health professionals (*n* = 9,472, 84.76%), which greatly increased the reliability of the ADE information. Nearly half of the outcomes were other serious outcomes (50.07%), followed by hospitalization, death, and life-threatening events, which occurred in 3,595 (27.92%), 1,440 (11.20%), and 934 (7.26%) cases, respectively.

Staphylococcal infections were the most commonly reported indication (*n* = 1,081, 9.67%), followed by TB (*n* = 673, 6.02%), infections (*n* = 552, 4.94%), pneumonia (*n* = 396, 3.54%), and Enterococcal infection (*n* = 355, 3.18%).

**FIGURE 3:**
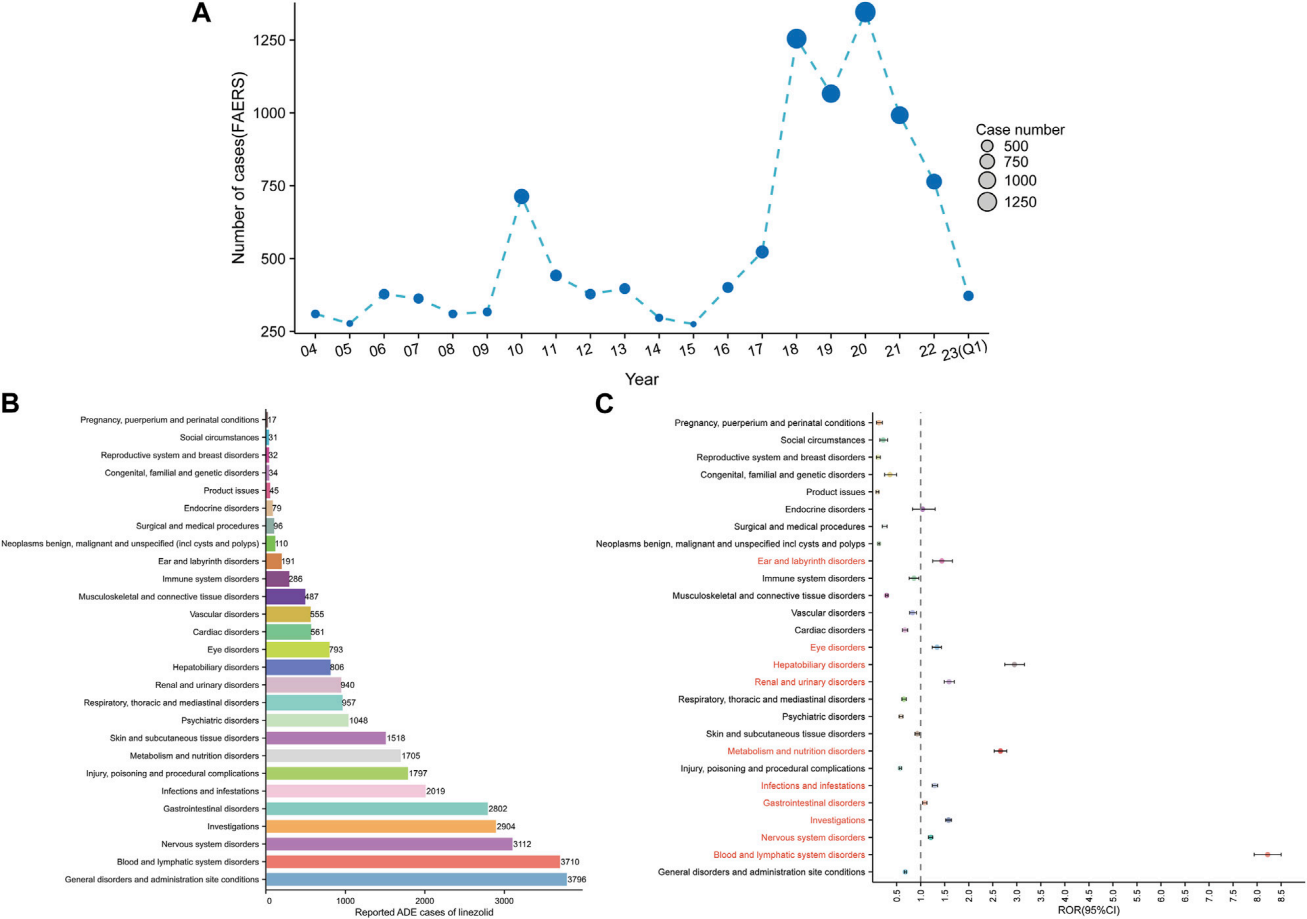
Signals detection at the SOC level. **(A)** Distribution of ADEs of linezolid from 2004 to the first quarter of 2023 (2023 Q1). **(B)** The bar chart displays the reported cases of ADEs at each SOC level. **(C)** Signals detection at the SOC level. The ROR values and their 95% confidence intervals (95% CI) are visualized. We label the SOCs with positive signal values to make a distinction. FAERS, Food and Drug Administration (FDA) Adverse Event Reporting System (FAERS); ADEs, adverse drug events; SOC, System Organ Class; ROR, reporting odds ratio.

Figure 3A shows the annual distribution of linezolid-related ADE reports. The lowest and highest number of reports were documented in 2015 (275 reports) and in 2020 (1346 reports), respectively. The number of ADE reports increased from 2015 to 2020 and remained high in 2020–2022.

### 3.2 Signal detects at the SOC level

Signal strengths and reports of linezolid at the SOC level are described in Supplementary Table S2. Linezolid-associated ADEs occurred in 27 organ systems. The number of case reports for linezolid-associated SOCs are shown in Figure 3B. The top five SOCs were general disorders and administration site conditions (*n* = 3,796, 12.47%), blood and lymphatic system disorders (*n* = 3,710, 12.19%), nervous system disorders (*n* = 3,112, 10.23%), investigations (*n* = 2,904, 9.54%), and gastrointestinal disorders (*n* = 2,802, 9.21%). Significant SOCs for which at least one of the four methods of disproportionality analysis met the criteria were blood and lymphatic system disorders (SOC code: 10005329, *n* = 3,710), nervous system disorders (SOC code: 10029205, *n* = 3,112), investigations (SOC code: 10022891, n = 2,904), gastrointestinal disorders (SOC code: 10017947, n = 2,802), infections and infestations (SOC code: 10021881, *n* = 2019), metabolism and nutrition disorders (SOC code: 10027433, *n* = 1705), renal and urinary disorders (SOC code: 10038359, *n* = 940), hepatobiliary disorders (SOC code: 10019805, *n* = 806), eye disorders (SOC code: 10015919, *n* = 793), and ear and labyrinth disorders (SOC code: 10013993, *n* = 191). Notably, disorders of the blood and lymphatic system were the SOCs that met all four criteria simultaneously (Supplementary Table S2). Figure 3C shows the ROR and its 95% confidence interval for linezolid-associated SOC signal strength.

### 3.3 Disproportionality analysis for ADEs associated with linezolid use

**FIGURE 4:**
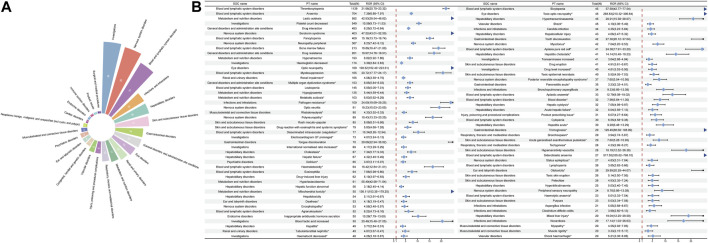
Signals detection at the PT level. **(A)** SOC attribution and number of 263 PTs that simultaneously satisfy the 4 methods of disproportionality analysis with positive signal values. **(B)** We selected PTs with a minimum of 20 cases and displayed the ROR and corresponding 95% CI using a forest plot. The blue arrows signify that the lower limit of the 95% confidence interval of the ROR exceeds 25.PTs, preferred terms; SOC, System Organ Class; PTs, preferred terms; CI, confidence interval; ROR, reporting odds ratio.

After excluding PT as a possible indication for linezolid medication, the 263 significantly disproportionate PTs corresponding to all four methods of disproportionality analysis simultaneously and ordered by the number of cases are displayed in Supplementary Table S3. Furthermore, we have ranked the SOCs in descending order according to the SOCs corresponding to these 263 PTs, as shown in Figure 4A. Next, we categorized PTs with more than 20 ADE cases and selected 93 ADEs that met this screening criterion, including 18 corresponding SOCs. To improve visualization, we present the PT signals in a forest plot format, arranged in descending order of case number (Figure 4B). Additionally, these data were grouped by SOC and the whole results are presented in Table 3. We identified that PT entries with more than 100 cases included thrombocytopenia (*n* = 1,139), anaemia (*n* = 704), lactic acidosis (*n* = 592), platelet count decreased (*n* = 549), drug interaction (*n* = 493), serotonin syndrome (*n* = 403), pancytopenia (*n* = 400), neuropathy peripheral (*n* = 367), bone marrow failure (*n* = 213), drug resistance (*n* = 201), hyponatraemia (*n* = 193), haemoglobin decreased (*n* = 174), optic neuropathy (*n* = 169), myelosuppression (*n* = 165), leukopenia (*n* = 145), and hypoglycaemia (*n* = 135), consistent with the medication warnings in the drug label. Interestingly, unexpected significant ADEs were identified, and PTs with more than 50 reports included renal impairment (*n* = 165), multiple organ dysfunction syndrome (*n* = 153), metabolic acidosis (*n* = 103), pathogen resistance (*n* = 100), rhabdomyolysis (*n* = 90), polyneuropathy (*n* = 89), drug reaction with eosinophilia and systemic symptoms (*n* = 79), disseminated intravascular coagulation (*n* = 77), electrocardiogram QT prolonged (*n* = 73), international normalised ratio increased (*n* = 69), hepatic failure (*n* = 67), delirium (*n* = 65), haematotoxicity (*n* = 64), mitochondrial toxicity (*n* = 55), deafness (*n* = 53), encephalopathy (*n* = 53), and agranulocytosis (*n* = 52). We also considered the IC025 value due to the increased stability of calculated results offered by the Bayesian approach in instances of low numbers of adverse events (Kubota et al., 2004). High IC025 values were found for unexpected signals such as toxic optic neuropathy (*n* = 45, IC025 [6.26]) and trichoglossia (*n* = 29, IC025 [5.23]), despite the low number of cases, indicating a strong association with linezolid administration. Digestive events, such as nausea, vomiting, and diarrhea were common in patients treated with linezolid. However, these events did not meet any of the four criteria set in our analysis.

**TABLE 3:** Signal strength of ADE reports for linezolid at the preferred term (PT) level in the FAERS database.

SOC name	PT name	Frequency	Case number	ROR (95% CI)	PRR	χ2	EBGM (EBGM05)	IC (IC025)
Blood and lymphatic system disorders	Sideroblastic anaemia	0.09%	27	517.55 (335.62–798.10)	517.09	10554.82	392.68 (273.30)	8.62 (6.93)
Blood and lymphatic system disorders	Bicytopenia	0.15%	45	57.58 (42.77–77.54)	57.5	2413.1	55.57 (43.33)	5.80 (4.13)
Blood and lymphatic system disorders	Aplasia pure red cell*	0.13%	41	24.38 (17.91–33.20)	24.35	904.55	24.01 (18.54)	4.59 (2.92)
Blood and lymphatic system disorders	Thrombocytopenia	3.74%	1139	21.98 (20.70–23.32)	21.19	21668.44	20.93 (19.91)	4.39 (2.72)
Blood and lymphatic system disorders	Myelosuppression	0.54%	165	20.72(17.77–24.17)	20.62	3041.86	20.37 (17.91)	4.35 (2.68)
Blood and lymphatic system disorders	Bone marrow failure	0.70%	213	18.86(16.47–21.60)	18.74	3537.31	18.54 (16.55)	4.21 (2.55)
Blood and lymphatic system disorders	Haematotoxicity*	0.21%	64	16.42 (12.84–21.01)	16.39	915.86	16.24 (13.21)	4.02 (2.35)
Blood and lymphatic system disorders	Pancytopenia	1.31%	400	15.16 (13.73–16.74)	14.97	5173.54	14.85 (13.67)	3.89 (2.23)
Blood and lymphatic system disorders	Aplastic anaemia*	0.11%	33	12.79 (9.08–18.02)	12.78	355.51	12.69 (9.52)	3.67 (2.00)
Blood and lymphatic system disorders	Disseminated intravascular coagulation*	0.25%	77	10.34 (8.26–12.94)	10.32	644.05	10.26 (8.50)	3.36 (1.69)
Blood and lymphatic system disorders	Blood disorder*	0.11%	32	7.98 (5.64–11.30)	7.97	194.25	7.94 (5.94)	2.99 (1.32)
Blood and lymphatic system disorders	Eosinophilia	0.21%	64	7.66 (5.99–9.80)	7.65	368.23	7.62 (6.20)	2.93 (1.26)
Blood and lymphatic system disorders	Anaemia	2.31%	704	7.39 (6.86–7.97)	7.25	3785.64	7.22 (6.78)	2.85 (1.19)
Blood and lymphatic system disorders	Cytopenia	0.10%	30	6.55 (4.58–9.38)	6.55	140.46	6.53 (4.83)	2.71 (1.04)
Blood and lymphatic system disorders	Agranulocytosis*	0.17%	52	6.22 (4.73–8.16)	6.21	226.31	6.19 (4.93)	2.63 (0.96)
Blood and lymphatic system disorders	Leukopenia	0.48%	145	5.95 (5.05–7.01)	5.93	592.45	5.91 (5.16)	2.56 (0.90)
Blood and lymphatic system disorders	Haemolytic anaemia*	0.08%	23	5.01 (3.33–7.54)	5.01	73.54	4.99 (3.55)	2.32 (0.65)
Blood and lymphatic system disorders	Lymphopenia	0.09%	26	3.85 (2.62–5.66)	3.85	54.74	3.84 (2.78)	1.94 (0.28)
Ear and labyrinth disorders	Ototoxicity*	0.09%	26	29.89(20.28–44.07)	29.87	712.42	29.35 (21.21)	4.88 (3.21)
Ear and labyrinth disorders	Deafness*	0.17%	53	4.18(3.19–5.47)	4.17	127.53	4.16 (3.32)	2.06 (0.39)
Endocrine disorders	Inappropriate antidiuretic hormone secretion	0.16%	50	10.29 (7.79–13.60)	10.28	416.22	10.22 (8.10)	3.35 (1.69)
Eye disorders	Toxic optic neuropathy*	0.15%	45	288.83 (210.32–396.64)	288.4	10948.67	245.15 (188.00)	7.94 (6.26)
Eye disorders	Optic neuropathy	0.56%	169	190.52 (162.40–223.51)	189.47	28381.15	169.82 (148.58)	7.41 (5.74)
Gastrointestinal disorders	Trichoglossia*	0.10%	29	129.46 (88.68–188.99)	129.34	3421.24	119.89 (87.36)	6.91 (5.23)
Gastrointestinal disorders	Tongue discolouration	0.23%	70	29.06 (22.94–36.82)	29	1859.12	28.51 (23.39)	4.83 (3.17)
Gastrointestinal disorders	Tooth discolouration	0.14%	42	27.30 (20.12–37.04)	27.26	1045.06	26.83 (20.78)	4.75 (3.08)
Gastrointestinal disorders	Pancreatitis acute*	0.12%	35	3.23 (2.32–4.51)	3.23	53.87	3.23 (2.45)	1.69 (0.02)
General disorders and administration site conditions	Drug resistance	0.66%	201	16.97 (14.76–19.51)	16.86	2970.08	16.70 (14.86)	4.06 (2.40)
General disorders and administration site conditions	Multiple organ dysfunction syndrome*	0.50%	153	6.85 (5.84–8.03)	6.82	757.72	6.80 (5.95)	2.77 (1.10)
General disorders and administration site conditions	Drug interaction	1.62%	493	6.25 (5.72–6.84)	6.17	2132.63	6.15 (5.71)	2.62 (0.95)
Hepatobiliary disorders	Hypertransaminasaemia	0.15%	45	20.91 (15.58–28.07)	20.88	841.12	20.63 (16.13)	4.37 (2.70)
Hepatobiliary disorders	Mixed liver injury*	0.07%	20	19.04 (12.25–29.59)	19.03	337.68	18.82 (13.01)	4.23 (2.57)
Hepatobiliary disorders	Hepatitis cholestatic*	0.13%	41	14.14 (10.40–19.23)	14.12	495.67	14.01 (10.83)	3.81 (2.14)
Hepatobiliary disorders	Hepatitis acute*	0.10%	30	9.28 (6.48–13.29)	9.27	220.12	9.22 (6.83)	3.21 (1.54)
Hepatobiliary disorders	Cholestasis*	0.22%	67	7.34 (5.77–9.33)	7.33	364.48	7.30 (5.97)	2.87 (1.20)
Hepatobiliary disorders	Hepatic cytolysis*	0.11%	32	7.05 (4.98–9.97)	7.04	165.15	7.01 (5.24)	2.81 (1.14)
Hepatobiliary disorders	Hepatotoxicity	0.18%	54	5.11 (3.91–6.67)	5.1	177.42	5.09 (4.07)	2.35 (0.68)
Hepatobiliary disorders	Drug-induced liver injury	0.20%	62	5.10 (3.97–6.55)	5.09	203.39	5.08 (4.12)	2.34 (0.68)
Hepatobiliary disorders	Acute hepatic failure*	0.11%	32	5.04 (3.56–7.13)	5.04	103.19	5.02 (3.76)	2.33 (0.66)
Hepatobiliary disorders	Hyperbilirubinaemia	0.08%	25	5.03 (3.40–7.45)	5.03	80.38	5.01 (3.61)	2.33 (0.66)
Hepatobiliary disorders	Hepatocellular injury	0.14%	43	4.68 (3.47–6.32)	4.68	124.02	4.67 (3.63)	2.22 (0.56)
Hepatobiliary disorders	Hepatic failure*	0.22%	67	4.32 (3.40–5.49)	4.31	169.94	4.30 (3.52)	2.10 (0.44)
Hepatobiliary disorders	Hepatitis*	0.16%	48	3.77 (2.84–5.01)	3.77	97.5	3.76 (2.97)	1.91 (0.25)
Hepatobiliary disorders	Hepatic function abnormal	0.18%	56	3.18 (2.45–4.14)	3.18	83.59	3.18 (2.55)	1.67 (0.00)
Infections and infestations	Pathogen resistance*	0.33%	100	24.00 (19.69–29.25)	23.93	2165.19	23.59 (20.00)	4.56 (2.89)
Infections and infestations	Nocardiosis	0.07%	20	17.14 (11.03–26.63)	17.13	300.55	16.96 (11.73)	4.08 (2.42)
Infections and infestations	Bronchopulmonary aspergillosis	0.11%	34	9.33 (6.66–13.08)	9.32	251.2	9.28 (6.99)	3.21 (1.55)
Infections and infestations	Aspergillus infection	0.07%	21	5.65 (3.68–8.67)	5.64	79.96	5.63 (3.93)	2.49 (0.83)
Infections and infestations	Candida infection	0.14%	44	4.35 (3.23–5.84)	4.34	112.9	4.33 (3.38)	2.12 (0.45)
Infections and infestations	Clostridium difficile colitis	0.07%	21	3.99 (2.60–6.13)	3.99	46.95	3.98 (2.78)	1.99 (0.33)
Injury, poisoning and procedural complications	Product prescribing issue*	0.10%	31	6.07 (4.27–8.64)	6.07	130.78	6.05 (4.50)	2.60 (0.93)
Investigations	Blood lactic acid increased	0.16%	50	20.46 (15.48–27.05)	20.43	912.58	20.19 (15.98)	4.34 (2.67)
Investigations	Platelet count decreased	1.80%	549	10.59 (9.73–11.53)	10.42	4652.28	10.36 (9.65)	3.37 (1.71)
Investigations	Drug level increased*	0.13%	40	4.81 (3.53–6.56)	4.81	120.26	4.80 (3.70)	2.26 (0.60)
Investigations	Haematocrit decreased*	0.15%	46	4.25 (3.18–5.67)	4.24	113.77	4.23 (3.32)	2.08 (0.42)
Investigations	International normalised ratio increased*	0.23%	69	4.17 (3.29–5.29)	4.17	165.67	4.16 (3.41)	2.06 (0.39)
Investigations	Electrocardiogram QT prolonged*	0.24%	73	4.07 (3.24–5.13)	4.07	168.51	4.06 (3.35)	2.02 (0.36)
Investigations	Transaminases increased	0.13%	41	3.64 (2.68–4.94)	3.64	78.19	3.63 (2.81)	1.86 (0.19)
Investigations	Haemoglobin decreased	0.57%	174	3.30 (2.84–3.83)	3.29	276.72	3.28 (2.90)	1.71 (0.05)
Metabolism and nutrition disorders	Mitochondrial toxicity*	0.18%	55	136.11 (103.35–179.25)	135.86	6795.77	125.47 (99.66)	6.97 (5.30)
Metabolism and nutrition disorders	Hyperlactacidaemia	0.20%	61	55.49 (42.98–71.64)	55.38	3150.37	53.59(43.28)	5.74 (4.08)
Metabolism and nutrition disorders	Lactic acidosis	1.95%	592	42.93 (39.54–46.62)	42.12	23174.56	41.08 (38.34)	5.36 (3.69)
Metabolism and nutrition disorders	Metabolic acidosis*	0.34%	103	6.83 (5.62–8.29)	6.81	508.53	6.78 (5.77)	2.76 (1.10)
Metabolism and nutrition disorders	Hyponatraemia	0.63%	193	6.82 (5.92–7.86)	6.78	948.73	6.76 (6.00)	2.76 (1.09)
Metabolism and nutrition disorders	Hypoglycaemia	0.44%	135	5.44 (4.59–6.44)	5.42	485.44	5.41 (4.69)	2.43 (0.77)
Musculoskeletal and connective tissue disorders	Myopathy*	0.07%	20	4.55 (2.93–7.05)	4.54	55.15	4.53 (3.14)	2.18 (0.51)
Musculoskeletal and connective tissue disorders	Rhabdomyolysis*	0.30%	90	4.33 (3.52–5.33)	4.32	229.37	4.31 (3.63)	2.11 (0.44)
Musculoskeletal and connective tissue disorders	Muscle rigidity*	0.07%	20	3.33 (2.15–5.17)	3.33	32.53	3.32 (2.30)	1.73 (0.07)
Nervous system disorders	Serotonin syndrome	1.32%	403	47.52 (43.01–52.50)	46.9	17601.38	45.61 (41.96)	5.51 (3.85)
Nervous system disorders	Optic neuritis	0.30%	91	19.23 (15.63–23.65)	19.17	1549.56	18.96 (15.95)	4.25 (2.58)
Nervous system disorders	Polyneuropathy*	0.29%	89	16.43 (13.33–20.25)	16.39	1273.16	16.23 (13.63)	4.02 (2.35)
Nervous system disorders	Peripheral sensory neuropathy	0.08%	24	8.76(5.86–13.08)	8.75	163.88	8.71 (6.22)	3.12 (1.46)
Nervous system disorders	Neuropathy peripheral	1.21%	367	8.23 (7.42–9.12)	8.14	2291.29	8.11 (7.44)	3.02 (1.35)
Nervous system disorders	Posterior reversible encephalopathy syndrome*	0.12%	37	7.65 (5.54–10.56)	7.64	212.51	7.61 (5.81)	2.93 (1.26)
Nervous system disorders	Myoclonus*	0.14%	42	7.04 (5.20–9.53)	7.03	216.27	7.00 (5.43)	2.81 (1.14)
Nervous system disorders	Status epilepticus*	0.09%	27	4.83 (3.31–7.04)	4.82	81.59	4.81 (3.51)	2.27 (0.60)
Nervous system disorders	Encephalopathy*	0.17%	53	4.56 (3.48–5.97)	4.55	146.65	4.54 (3.63)	2.18 (0.52)
Psychiatric disorders	Delirium*	0.21%	65	3.97 (3.11–5.07)	3.96	143.79	3.96 (3.23)	1.98 (0.32)
Renal and urinary disorders	Tubulointerstitial nephritis*	0.16%	48	4.87 (3.67–6.47)	4.87	147.08	4.86 (3.83)	2.28 (0.61)
Renal and urinary disorders	Renal impairment*	0.54%	165	4.08 (3.50–4.75)	4.06	380.3	4.05 (3.57)	2.02 (0.35)
Respiratory, thoracic and mediastinal disorders	Tachypnoea*	0.09%	28	4.33 (2.99–6.27)	4.33	71.43	4.32 (3.17)	2.11 (0.44)
Respiratory, thoracic and mediastinal disorders	Bronchospasm*	0.10%	29	3.94 (2.74–5.67)	3.94	63.43	3.93 (2.90)	1.97 (0.31)
Skin and subcutaneous tissue disorders	Hypersensitivity vasculitis	0.09%	28	18.18 (12.52–26.38)	18.16	449.02	17.97 (13.16)	4.17 (2.50)
Skin and subcutaneous tissue disorders	Rash maculo-papular	0.28%	85	8.06 (6.51–9.98)	8.04	521.86	8.01 (6.70)	3.00 (1.34)
Skin and subcutaneous tissue disorders	Acute generalised exanthematous pustulosis*	0.10%	29	7.60 (5.28–10.95)	7.59	165.31	7.56 (5.57)	2.92 (1.25)
Skin and subcutaneous tissue disorders	Drug reaction with eosinophilia and systemic symptoms*	0.26%	79	5.83 (4.68–7.28)	5.82	314.54	5.81 (4.82)	2.54 (0.87)
Skin and subcutaneous tissue disorders	Toxic epidermal necrolysis	0.13%	40	5.52 (4.05–7.53)	5.51	147.35	5.50 (4.24)	2.46 (0.79)
Skin and subcutaneous tissue disorders	Toxic skin eruption	0.09%	26	5.14 (3.50–7.56)	5.14	86.35	5.12 (3.71)	2.36 (0.69)
Skin and subcutaneous tissue disorders	Purpura	0.08%	23	5.03 (3.34–7.58)	5.03	74.08	5.02 (3.56)	2.33 (0.66)
Skin and subcutaneous tissue disorders	Petechiae	0.09%	26	4.93 (3.35–7.24)	4.92	81.03	4.91 (3.56)	2.30 (0.63)
Skin and subcutaneous tissue disorders	Drug eruption	0.13%	41	4.91 (3.61–6.67)	4.9	127.08	4.89 (3.78)	2.29 (0.62)
Vascular disorders	Shock haemorrhagic*	0.07%	20	5.21 (3.36–8.08)	5.2	67.72	5.19 (3.59)	2.38 (0.71)
Vascular disorders	Shock*	0.15%	45	4.10 (3.06–5.49)	4.09	104.99	4.09 (3.20)	2.03 (0.36)

The table demonstrates the 93 PT entries that satisfy all 4 methods of disproportionality analysis with positive signal values and a number of cases not less than 20. PT entries are categorized by SOC. Asterisks (*) indicate unexpected signals that are not indicated in the drug label. ADE, adverse drug event.

Additionally, given the potential confounding effect of baseline information on the results of the disproportionality analyses (de Vries et al., 2020), sensitivity analyses incorporating weight (<80 kg, 80–100 kg, >100 kg), age (18–64 years, >64 years, subgroups <18 years were under-reported and excluded), gender (male, female), and reported population (consumers and health professionals) were performed to bolster result confidence (Liang et al., 2023). Notably, serotonin syndrome (*n* = 18, ROR 46.07, 95% CI 28.93–73.37) exhibited the highest signal strength in the group >100 kg; however, it was absent from the first 15 adverse drug event signals in the two groups ≤100 kg. Moreover, other signals exclusive to the group >100 kg comprised asthenia (*n* = 20, ROR 2.39), sepsis (*n* = 15, ROR 6.00), product use issue (*n* = 11, ROR 2.81), and paraesthesia (*n* = 11, ROR 3.01) (Supplementary Figure S1). Similar sensitivity analyses were conducted to assess the impact of age (Supplementary Figure S2), gender (Supplementary Figure S3), and reported person (Supplementary Figure S4) on the signals within distinct subgroups. This critical assessment provides essential insights into refining clinical management strategies, enabling clinical decision-makers to customize treatments based on the specific characteristics of these subgroups.

### 3.4 Gender-based difference in risk signals for linezolid

**FIGURE 5:**
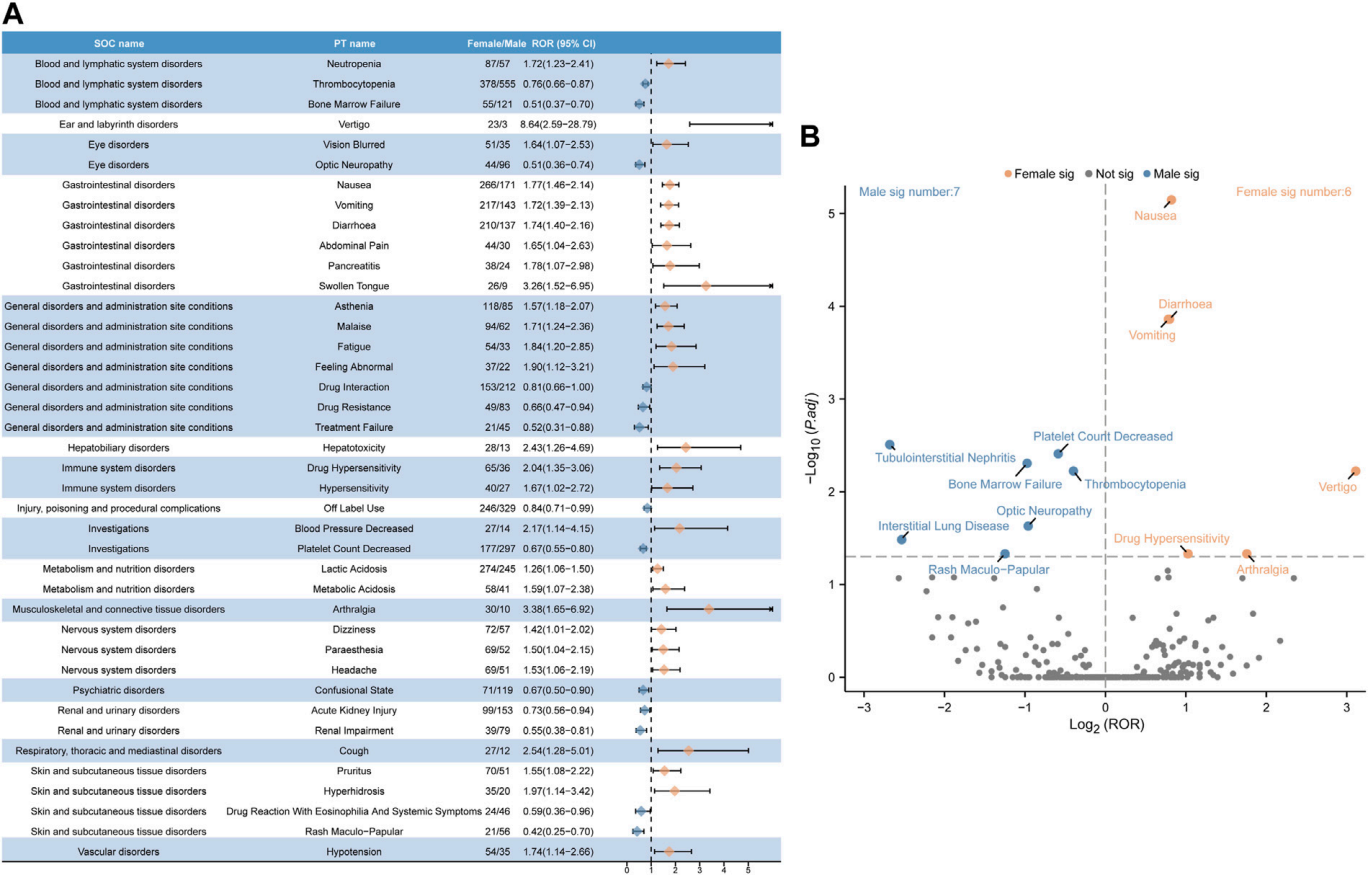
Analysis of gender-differentiated risk signals in linezolid. **(A)** Reporting odds ratios (ROR) with 95% CI for all positive gender-related ADEs. **(B)** Gender-differentiated risk signal volcano plot for linezolid. The horizontal coordinate shows the log2 ROR value and the vertical coordinate indicates the adjusted *p*-value after -log10 conversion. Significant signals are highlighted and annotated in prominent colors. The *p*-value is adjusted with false discovery rate (FDR) method.

The distribution of linezolid volume is slightly lower in females than in males, and plasma concentrations of the drug are higher in females than in males(Sisson et al., 2002). However, a significant increase in drug exposure in females above known, well-tolerated levels is unexpected. To analyze whether gender influences linezolid adverse effects, we used the ROR method to identify 40 PTs with disproportionate ADE incidence between males and females, categorized by SOC. The results are presented in Figure 5A. The results of all data are presented in Supplementary Table S4. Some ADEs such as thrombocytopenia, bone marrow failure, optic neuropathy, drug interaction, drug resistance, treatment failure, off-label use, decrease in platelet count, state of confusion, acute kidney injury, renal impairment, drug reaction with eosinophilia and systemic symptoms, and rash maculo-papular were more common in males. High-risk ADEs in females included neutropenia, vertigo, vision blurred, nausea, vomiting, diarrhea, abdominal pain, pancreatitis, swollen tongue, asthenia, malaise, fatigue, feeling abnormal, hepatotoxicity, drug hypersensitivity, blood pressure decreased, lactic acidosis, arthralgia, dizziness, paresthesia, headache, cough, pruritus, hyperhidrosis, and hypotension.

We plotted a “volcano diagram” to visualize the signals and analyze the results of gender-based differences in ADE signal extraction for linezolid (Figure 5B). Each point in the figure represents a linezolid-associated ADE and we labeled statistically significant ADEs. Seven significant signals were observed in males, including tubulointerstitial nephritis, platelet count decrease, bone marrow failure, thrombocytopenia, optic neuropathy, interstitial lung disease, and rash maculo-papular. Six adverse reactions were observed in females, including nausea, diarrhea, vomiting, vertigo, drug hypersensitivity, and arthralgia.

### 3.5 TTO analysis of linezolid-related ADEs from overall and subgroup perspectives

After excluding inaccurate, missing, or unknown reports of onset, 4,362 ADEs were collected, and the median TTO was determined as 6 days (interquartile range [IQR] 1–15 days) (Supplementary Table S5).

**FIGURE 6:**
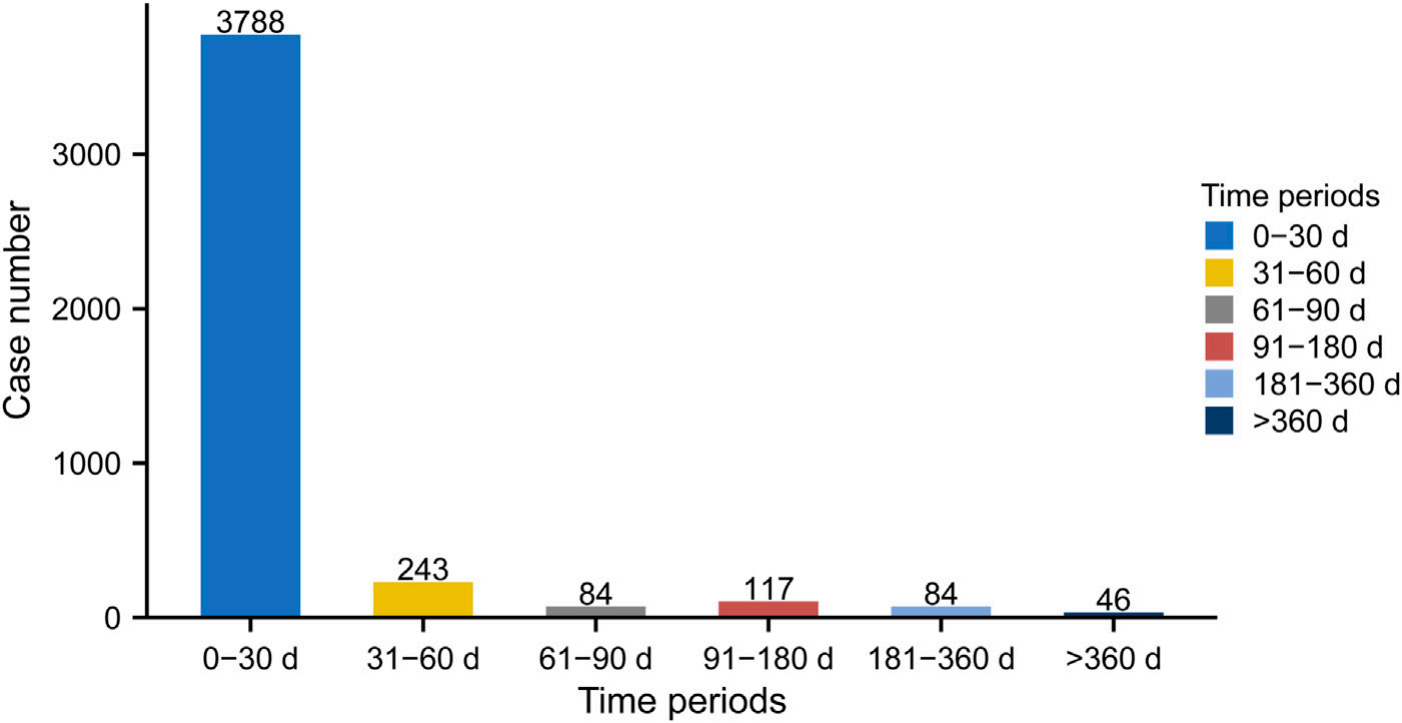
Time to onset (TTO) analysis (counted in days) of linezolid-related ADEs at the overall level. The frequency bar chart illustrates the distribution of TTO reports across various time periods.

As shown in Figure 6, most cases occurred within the first month (*n* = 3,788, 86.84%) of linezolid administration. The number of ADEs decreased over time, with 243 ADEs (5.57%) occurring in the second month and 84 ADEs (1.93%) in the third month. Notably, in 1.05% of cases, adverse drug events could still occur even after 1 year of treatment with linezolid. To examine whether the risk of linezolid-associated ADEs increases or decreases over time, we conducted Weibull distribution tests on both the overall patient population and various subgroups. For overall analysis, the calculated shape parameter (β) was 0.62 and the upper limit of its 95% confidence interval (CI) was 0.64. Both values were below 1, indicating a decline in the prevalence of ADEs over time (Supplementary Table S5). In the subgroup analyses based on age, it is noteworthy that β values were close to 1 for the subgroups <18 years (*n* = 6, β 2.53, 95% CI 0.51–4.55) and >64 years (*n* = 70, β 1.06, 95% CI 0.86–1.27). Additionally, their 95% CI encompassed 1, indicating the Weibull curve type as random failure, and implying a continued occurrence of ADEs over time. Additionally, the Weibull distribution test for the remaining subgroups revealed that all curve types were early failure. Comprehensive statistical descriptions for the different subgroups of TTO could be found in Supplementary Table S5.

### 3.6 TTO analysis of linezolid-related ADEs at the SOC and PT levels

**FIGURE 7:**
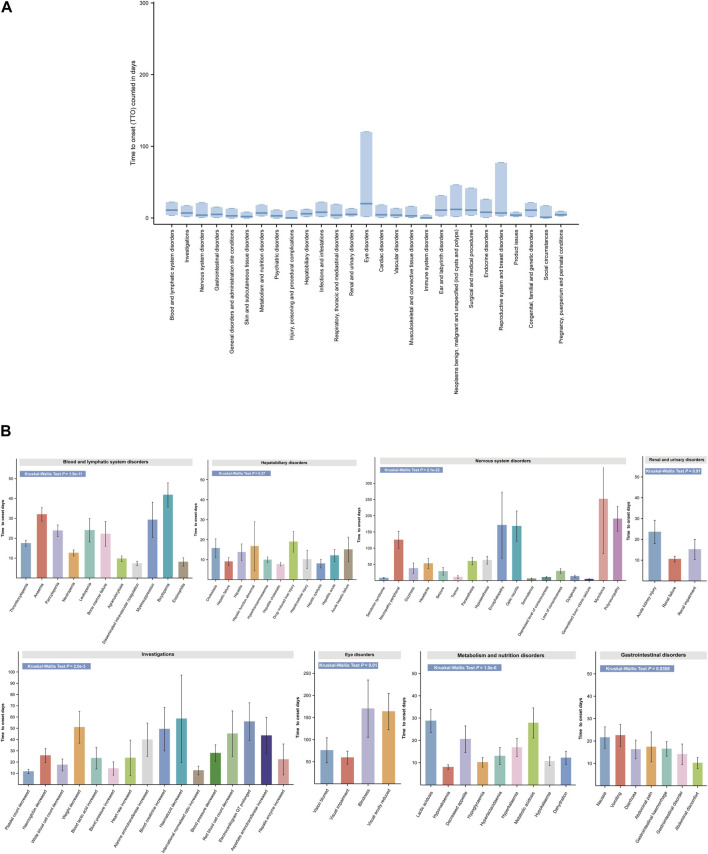
Time to onset (TTO) analysis of ADEs at the SOC and PT levels. **(A)** Box plot of the TTO at the SOC level for linezolid. Bold bar within the stick: median TTO; Lower end of the stick: 1/4 quantile of the TTO; Upper end of the stick: 3/4 quantile of the TTO. **(B)** Specific comparison of TTO in PTs at eight different SOC levels. SOC, System Organ Class; PTs, preferred term.

ADEs in clinical trials for linezolid have focused on single or limited number of organ systems. To determine the timing of ADEs in more detail, we analyzed the TTO at the SOC level (Figure 7A). Linezolid-related eye disease, the median longest-onset SOC, occurred at a median of 20 days (IQR 2–120 days). In contrast, injury, poisoning, and procedural complications (IQR 0–10 days) and immune system disorders (IQR 0–4 days) had the shortest median disease onset times associated with linezolid, each at 0 days. Other systemic diseases, such as blood and lymphatic system disorders, infections and infestations, ear and labyrinth disorders, neoplasms benign, malignant, and unspecified, surgical and medical procedures, endocrine disorders, and congenital, familial, and genetic disorders, had a median time to onset of 1–2 weeks. The median onset of most adverse events, including the other 19 SOCs, was within 1 week (Supplementary Table S6).

SOCs often contain multiple types of PTs. For clarity on the onset time of individual PTs in the SOC and to identify whether there are differences in the onset time of PTs within the same SOC, we analyzed and compared the detailed onset times of ADEs at the PT level according to the SOC (Figure 7B and Supplementary Table S6). Overall, except for liver disease (*p* = 0.37) and kidney disease (*p* = 0.91), there was a significant difference in TTO between PTs for the remaining six SOCs (*p* < 0.05). The mean (standard deviation [SD]) time for the earliest occurrence of disseminated intravascular coagulation in hematologic and lymphatic disorders was 7.45 days (7.30 days), while the average time for the latest occurrence of bicytopenia was 42.5 days (34.65 days). More detailed and complete results are shown in Figure 7B and Supplementary Table S6. These TTO analyses at the SOC and PT levels provide a more precise guide for detecting adverse events following linezolid administration.

## 4 Discussion

Our findings show that linezolid-related side effects occur more frequently in males (46.37%) than in females (36.04%). This can be attributed to the main indications of linezolid such as staphylococcal infection, tuberculosis, infection, pneumonia, etc., which are more common in males (Shaweno et al., 2021; Corica et al., 2022; Stensen et al., 2022). Epidemiological characteristics of the population support our results. Unfortunately, most reports (97.15%) do not contain detailed information on the patients’ age. Overweight and obese patients have lower linezolid exposure rates and appear to be at higher risk of treatment failure (Bandín-Vilar et al., 2022). Given this background, we conducted a study stratified by population weight (Supplementary Figure S1). We found that unique signals, such as asthenia (*n* = 20, ROR 2.39) and serotonin syndrome (*n* = 18, ROR 46.07), were discovered among the top 15 significant signals in a subgroup of patients weighing >100 kg. Due to the lack of specific dosages of medications used by patients and the lack of body weight data for almost 70% of patients, our results can only be considered indicative, but serious neurological side effects should be of particular concern within this patient subgroup. Nearly, 84.76% of adverse event reports were documented by healthcare professionals. Notably, serious adverse outcomes such as hospitalization, death, and life-threatening conditions accounted for approximately half of linezolid-related outcomes (46.38%). The annual distribution of linezolid-related ADE reports showed an annual increase in linezolid-related ADE reports since 2015 (Figure 3A). These results highlight the widespread clinical use and efficacy of linezolid and emphasize the importance of improving the detection of linezolid-related adverse drug reactions for its association with serious adverse events in clinical settings.

Based on the results of disproportionality analysis, significant signals at the SOC level were indicated for blood and lymphatic system disorders, nervous system disorders, investigations, gastrointestinal disorders, infections and infestations, metabolism and nutrition disorders, renal and urinary disorders, hepatobiliary disorders, eye disorders and ear and labyrinth disorders (Figure 3C). In addition to ear and labyrinth diseases, some SOCs were commonly reported in clinical trials and mentioned in the drug label (Wunderink et al., 2012; Tang et al., 2015; O'Riordan et al., 2019; Wunderink et al., 2021). Several specific adverse reactions mentioned in the drug label such as myelosuppression, peripheral and optic neuropathy, serotonin syndrome, increased blood pressure, lactic acidosis, hypoglycemia, and drug resistance were found to be positive signals in the present study, further confirming the reliability of our results.

Of the significant SOC signals, the most common were indicated for blood and lymphatic system disorders, nervous system disorders, investigations, gastrointestinal disorders, metabolism, and nutrition disorders. However, SOCs with a small number of cases also showed significant signals, for example, in diseases of the ear and labyrinth (*n* = 191).

### 4.1 ADEs related to the disorders of the blood and lymphatic system

The three most common ADEs in the blood system in terms of report numbers were thrombocytopenia (*n* = 1,139, ROR 21.98 [20.70–23.32]), anemia (*n* = 704, ROR 7.39 [6.86–7.97]), and pancytopenia (*n* = 400, ROR 15.16 [13.73–16.74]). Numerous previous clinical studies have confirmed linezolid’s hematological toxicity. In a double-blind, randomized, controlled trial in drug-resistant TB, different doses of linezolid resulted in myelosuppression in approximately 11.6% of patients (Conradie et al., 2022). In the Nix-TB phase 3 trial (NCT02333799), adverse events above grade 1, including thrombocytopenia (6%) and anemia (37%), severely limited the use of linezolid (Conradie et al., 2020). In a randomized, double-blind, phase 3 trial of two-week linezolid for the treatment of hospital- or ventilator-acquired pneumonia, the incidence of treatment-emergent adverse events, thrombocytopenia, and anemia related to linezolid therapy was 0.8% and 1.1%, respectively (Wunderink et al., 2021). A meta-analysis that summarized the results of 11 randomized controlled trials of skin and soft tissue infections showed that patients using linezolid were more likely to develop thrombocytopenia than those on vancomycin (Li and Xu, 2018). However, the mechanism of linezolid-induced hematological toxicity largely remains unknown. One possible molecular mechanism is that linezolid leads to the increased phosphorylation of myosin light chain 2, which further regulates platelet release in MEG-01 cells (Tajima et al., 2016). Furthermore, Wang et al. observed that linezolid-induced thrombocytopenia was associated with reduced antioxidant capacity as well as lipid peroxidation and free radical formation (Wang et al., 2016). Severe thrombocytopenia is immunologically related, and a study found the presence of linezolid-associated platelet antibodies in thrombocytopenic patients (Pascoalinho et al., 2011). Importantly, risk factors for linezolid-associated thrombocytopenia include baseline platelet count, minimum concentration, and renal insufficiency, which must be considered when administering linezolid (Matsumoto et al., 2010; Natsumoto et al., 2014; Tsuji et al., 2017; Crass et al., 2019; Cazavet et al., 2020). Through a real-world analysis of linezolid, we also identified several significant hematologic adverse signals, including bone marrow failure (*n* = 213, ROR 18.86 [16.47–21.60]), myelosuppression (*n* = 165, ROR 20.72 [17.77–24.17), leukopenia (*n* = 145, ROR 5.95 [5.05–7.01]), eosinophilia (*n* = 64, ROR 7.66 [5.99–9.80]), and bicytopenia (*n* = 45, ROR 57.58 [42.77–77.54]), consistent with previous clinical studies and drug label. We also identified new and unexpected signals, such as disseminated intravascular coagulation (DIC) (*n* = 77, ROR 10.34 [8.26–12.94]), aplasia pure red cell (*n* = 41, ROR 24.38 [17.91–33.20]) and aplastic anaemia (*n* = 33, ROR 12.79 [9.08–18.02]). DIC is associated with disease progression and portends poor outcomes. This could be related to the other side effects of linezolid found in our data, such as thrombocytopenia, a hypocoagulable state, and low fibrinogen levels. Cases of aplasia pure red cell caused by linezolid have been reported (Monson et al., 2002; Waki et al., 2012; Yang et al., 2022), which is consistent with our results. In summary, in addition to hemoglobin and platelet count, coagulation and reticulocyte count must also be considered. When using anticoagulants and antiplatelet agents in patients, linezolid should be used with caution.

### 4.2 ADEs related to nervous system disorders

Peripheral neuropathy and optic neuropathy are common neurological adverse reactions underlying the main reason for the discontinuation of linezolid (Rubinstein et al., 2001; Koh et al., 2012; Tang et al., 2015; O'Riordan et al., 2019). However, our real-world data showed that the most frequent case report of neurological adverse effects was serotonin syndrome (*n* = 403, ROR 47.52 [43.0–52.50]). This may be due to the different identities of the reporters, leading to different descriptions of peripheral neuropathy and optic neuropathy in general. However, relative to the total number of cases, peripheral neuropathy and optic neuropathy remain the most common, which is consistent with clinical observational studies. Serotonin syndrome is a rare but potentially fatal adverse drug reaction (Boyer and Shannon, 2005; Woytowish and Maynor, 2013). The mechanism by which linezolid causes serotonin syndrome may be that it does not selectively inhibit the enzyme monoamine oxidase, resulting in serotonin overload in the central nervous system (Francescangeli et al., 2019). Therefore, linezolid may interact with other medications and increase the risk of serotonin syndrome (Gatti et al., 2021b). According to recent studies, including a cohort study (Bai et al., 2022), a cross-sectional study (Traver et al., 2022), and a retrospective cohort study (Kufel et al., 2023), serotonin syndrome is a rare linezolid-induced ADE, whereas it was reported in larger numbers in our study. This result suggests that vigilance should be exercised toward linezolid-induced serotonin syndrome and further studies are required to assess the risk. Several clinical trials have shown that the long-term use of linezolid predisposes patients to peripheral neuropathy. In ZeNix’s study, grade 3 or lower peripheral neuropathy was reported in 45 of 181 participants (25%) across all groups (Conradie et al., 2022). Investigator-reported peripheral neuropathy (≥ Grade 1) was reported in 80 (77%) participants (Imperial et al., 2022). Eighty-eight (81%) participants developed peripheral neuropathy at the time of treatment, typically with mild to moderate symptoms (Conradie et al., 2022). Side effects of optic neuritis were also reported in the clinical studies mentioned above. We identified some PTs with positive signal values but relatively small numbers, such as myoclonus (*n* = 42, ROR 7.04 [5.20–9.53]), posterior reversible encephalopathy syndrome (n = 37, ROR 7.65 [5.54–10.56]), status epilepticus (*n* = 27, ROR 4.83 [3.31–7.04]); these were not highlighted in the drug label. Although peripheral neuropathies are mentioned in the drug label, our data provide a detailed list of case numbers and signal values of linezolid-associated neurological adverse effects. These provide clues for the doctors to promptly recognize this side effect.

Adverse drug reactions associated with ocular lesions complicate long-term treatment with linezolid. As shown in Table 3, optic neuropathy shows both a high number of reported cases (*n* = 169) as well as a higher signal value (ROR 190.52, PRR 189.47, EBGM05 148.58, IC025 5.74). Optic neuropathy associated with linezolid has been reported in many clinical trials. A French clinical trial for multidrug-resistant tuberculosis reported a high incidence of confirmed optic neuropathy (25% of the cohort) (Jaspard et al., 2020). Two meta-analyses on the safety of linezolid use in drug-resistant TB reached similar conclusions (Sotgiu et al., 2012; Lifan et al., 2019). The above findings support our results. However, a synthesis of the results from the two prospective studies found no optic neuropathy in children with multidrug-resistant TB taking linezolid (Garcia-Prats et al., 2019). The inconsistency in these findings may be related to the different baseline characteristics of the included populations and the short follow-up period. Toxic optic neuropathy (*n* = 45, ROR 288.83 [210.32–396.64]) is also a specific manifestation of optic neuropathy. Some case reports suggest that this ADE may develop rapidly (Lee et al., 2003; Joshi et al., 2009). Taken together, patients administered linezolid need a regular, periodic eye exam to detect possible optic neuropathy in the early stage.

### 4.3 ADEs related to gastrointestinal disorders

Among the ADEs of the gastrointestinal system, some PTs that were not indicated in the drug label were found. Trichoglossia (*n* = 29, ROR 129.46 [88.68–188.99]), or black hairy tongue (BHT), is characterized by pigmentation, whereby the dorsal tongue appears black, green, or yellow; it is a self-limiting benign disease. Linezolid-associated trichoglossia has primarily been documented in case reports (Jover-Diaz et al., 2010; Lee et al., 2021; Tomita et al., 2023). A literature report of three patients who developed black hairy tongue after treatment with linezolid all demonstrated severe dysbiosis in their oral bacterial communities, with Proteobacteria being the most common phylum, suggesting that linezolid may cause disruption of the oral flora (Shangguan et al., 2022). Acute pancreatitis (*n* = 35, ROR 3.23 [2.32–4.51]) is an unexpected adverse effect. In several cases it has been reported that linezolid can cause acute pancreatitis (Fortún et al., 2005; Rose et al., 2012; Kim et al., 2023). However, the exact mechanism of occurrence is unknown. This may be due to mitochondrial dysfunction resulting in damage to the pancreatic acinar cells, but this requires further validation. Our data also reported adverse reactions consistent with those specified on the drug label, such as tongue discoloration (*n* = 70, ROR 29.06 [22.94–36.82]) and tooth discoloration (*n* = 42, ROR 27.30 [20.12–37.04]). A high incidence of vomiting and diarrhea has been reported in several previous studies (Li and Xu, 2018; Shi et al., 2023), but no positive signals were found after our disproportionality analysis. This may be because these adverse reactions are common with the adverse reaction reports for other drugs in the FAERS database, which in turn influences the signal value. Disproportionality requires a higher (or lower) frequency of ADE reporting for certain drugs. The absence of a signal does not mean that there are no relative adverse events but simply indicates that these side effects are not disproportionately common.

### 4.4 ADEs at other SOC levels

The results of our disproportionality analysis suggested that ADEs associated with linezolid medications may also affect other organs or tissues. Renal diseases associated with linezolid dosing observed in our study included renal impairment (*n* = 165, ROR 4.08 [3.50–4.75]) and tubulointerstitial nephritis (*n* = 48, ROR 4.87 [3.67–6.47]), with a greater number of case reports and positive signals. According to the drug label, no dosage adjustments are recommended for patients at any stage of renal impairment, including hemodialysis. The trough concentration of linezolid increases due to renal dysfunction, resulting in an increased incidence of adverse reactions (Bandín-Vilar et al., 2022; Wu et al., 2022). A report described a case of a patient presenting with drug rash with eosinophilia and systemic symptoms indicative of linezolid-associated acute interstitial nephritis (Savard et al., 2009). Although no dose adjustments are recommended in the drug label for patients with renal impairment, we recommend that physicians be aware of these possible side effects and continuously monitor renal function when linezolid is administered.

Moreover, we identified unexpected significant safety signals for rhabdomyolysis (*n* = 90, ROR 4.33 [3.52–5.33]), electrocardiogram QT prolonged (*n* = 73, ROR 4.07 [3.24–5.13]) and cholestasis (*n* = 67, ROR 7.34 [5.77–9.33]). Rhabdomyolysis is a potentially life-threatening disease caused by damage to muscle cells and the subsequent release of cellular components into the bloodstream (Stahl et al., 2020). Four cases of rhabdomyolysis associated with linezolid have been reported (Allison et al., 2009; Carroll et al., 2012; Lechner et al., 2017). Moreover, a FAERS-based study identified an association between linezolid and prolongation of the QT interval along with a higher incidence in TB patients (Shao et al., 2023). The causal relationship between linezolid and rhabdomyolysis and QT interval prolongation is unclear, necessitating further clinical studies to comprehend the pathogenesis of these adverse events. In conclusion, our findings present a comprehensive list of linezolid side effects across various SOCs, which serves as a valuable reference for physician decision-making. Physician awareness of these novel and unexpected signals is crucial. If deemed necessary, the FDA can update the drug label and release appropriate warnings.

### 4.5 Gender-based differences

As described previously, in the description of the baseline profile, we found proportional differences in the gender distribution of ADEs. In fact, the analysis of gender differences must be considered when assessing drug safety, which facilitates more precise management of ADEs. The study found that males are more prone to develop tubulointerstitial nephritis, platelet count decreased, bone marrow failure, thrombocytopenia, optic neuropathy, interstitial lung disease, and rash maculo-papular, while females are more prone to suffer from nausea, diarrhoea, vomiting, vertigo, drug hypersensitivity, arthralgia. Of note, thrombocytopenia and platelet count decrease are common in both males and females, but males are at higher risk of develop hematologic ADEs, while females exhibited a greater association with gastrointestinal disorders in comparison to males. Gastrointestinal-related symptoms (nausea, vomiting, and diarrhea) were the most common reasons for discontinuation of the drug (Moise et al., 2002; Noel et al., 2012). Nausea (Shorr et al., 2015), gastrointestinal intolerance (Veerman et al., 2023), diarrhea, and vomiting (Shi et al., 2023) were the most common side effects reported in previous studies. A single-center retrospective observational study (Tsutsumi et al., 2022) found that female gender is an independent risk factor for linezolid-induced vomiting, which may be associated with multiple signaling pathways including D2, 5-HT3, and neurokinin-1 receptors (Navari and Aapro, 2016). However, in addition to gender-related biological factors, gendered social factors are primarily responsible for this gender difference. Males tend to downplay illness (i.e., wait for symptoms to subside without intervening) compared to females, who engage in more active health-promoting behaviors and have frequent contact with healthcare professionals, which may contribute to male’s likelihood of more serious illnesses adverse events is higher (Lee et al., 2023). Our results provide these gender-specific side effects. Although these findings require subsequent validation, they offer improved guidance on medication monitoring for both males and females. In addition, we should emphasize gendered social factors in order to better improve the safe use of linezolid.

### 4.6 TTO analysis

The temporal relationship between administration and time of onset is crucial for assessing drug safety as it identifies specific risk windows and leads to prevention or early diagnosis of adverse reactions (Leroy et al., 2014). Our findings indicated that adverse reactions associated with linezolid mostly occurred in the first 2 months (92.41%), with the highest incidence in the first month (*n* = 3,788, 86.84%), followed by the second month (*n* = 243, 5.57%). The main indications for linezolid are infections, including staphylococcal infection, infection, pneumonia and enterococcal infection, in which linezolid is not used for a long period of time, which may result in the vast majority of adverse drug reactions being concentrated in the first month, and in general we tend to associate side effects with the start of treatment with the drug, which can lead to biased results. The Weibull distribution test revealed a decrease in the probability of ADEs over time within the general population and across most subgroups. However, ADEs persisted over time in subgroups comprising individuals younger than 18 years and those older than 64 years. This indicates that continued vigilance for ADEs related to linezolid is especially warranted in both subgroups. Most importantly, we provide a comprehensive study of the specific time of onset following linezolid administration in each organ system. Previous researchers have reported the median time to occurrence of some specific ADEs for linezolid dosing (Matsumoto et al., 2014; Jaspard et al., 2020; Veerman et al., 2023). Although these results may not reflect the actual TTO given the population in the clinical trial, our results are close to the findings and provide a more detailed and concrete list. Abnormal liver function tests are among the side effects listed in the drug label of linezolid. Our data analysis revealed that PT associated with hepatobiliary disease occurred on average within 1–3 weeks. Linezolid can cause many types of neurological side effects, some of which occur on average after more than 4 months, e.g. neuropathy peripheral, encephalopathy, optic neuritis, myoclonus, and polyneuropathy. The effects of linezolid on the urinary system include acute kidney injury, renal failure, and renal dysfunction, which occur on average within 1 month. Eye disorders are serious side effects of linezolid use, and can cause blurred vision, visual impairment, blindness, and even vision loss, with the first two typically occurring earlier than the latter two, at about 2 months, and the latter two on average more than 5 months later. Severe gastrointestinal symptoms are the main reasons for discontinuation of linezolid, and these reactions often occur within 3 weeks or less. The median TTO was less than 2 months for all systems, except neurological and ocular adverse events. Therefore, the requirements for early detection and follow-up of adverse reactions occurring in different systems should be different. Our research makes a valuable contribution in this area by helping to better and promptly reduce patient discomfort and improve the patient’s experience with medication outcomes.

Other studies have reported associations between linezolid and specific side effects using centralized data (Lee and Caffrey, 2018; Dai et al., 2020; Gatti et al., 2021a; Battini et al., 2022; Ni et al., 2022; Shao et al., 2023). This study, for the first time, comprehensively documented and evaluated the safety of post-marketing administration of linezolid based on the largest sample of real-world data to date. However, some limitations remain. Firstly, the FAERS is inherently limited by underreporting, incomplete reporting, and selective reporting (Alatawi and Hansen, 2017; Raschi et al., 2019). Less serious or common adverse events may be underreported, while more serious or rare events may be overreported (Wang et al., 2023). Among the ADE reports we collected, 97% were missing detailed age data, and 85% originated from health professionals. Therefore, potential biases caused by the data should be carefully considered when interpreting the results (Shi et al., 2023). Secondly, the absence of detailed clinical information on patients such as comorbidities, severity of underlying disease, and concomitant medications, further hinders the control for confounding variables (Zhang et al., 2023). Thirdly, disproportionality analyses are limited to assessing signal strength and establishing statistical associations; they are unable to quantify risk or determine causality (Ji et al., 2022b). Fourth, because the total number of people receiving linezolid administration is unknown, we were unable to quantify the incidence of each ADE (Chedid et al., 2018). Despite these limitations, inherent in the use of the FAERS database for pharmacovigilance studies, a comprehensive characterization of ADEs related to linezolid in this study may provide insightful evidence for safe use and further clinical studies.

## 5 Conclusion

In conclusion, our comprehensive and systematic pharmacovigilance analysis of the FAERS database identified several common and rare side effects of linezolid use and their associated timing. Careful monitoring of all populations and determination of the risk of these adverse reactions is recommended. Despite offering valuable evidence for the safety of linezolid, our study necessitates meticulous consideration of the inherent limitations of the FAERS database, as well as potential confounders and biases. This calls for a more cautious interpretation of our analysis results. Additionally, future prospective clinical trials and epidemiological studies will provide a more precise evaluation of the safety risks associated with linezolid.

## Data Availability

The original contributions presented in the study are included in the article/Supplementary Material, further inquiries can be directed to the corresponding author.

## Supplementary material

SUPPLEMENTARY FIGURE S1 The top 15 adverse event signals at the preferred term level, stratified by weight groups. The arrows signify that the lower limit of the 95% confidence interval of the ROR exceeds 25.

SUPPLEMENTARY FIGURE S2 The top 15 adverse event signals at the preferred term level, stratified by age groups.

SUPPLEMENTARY FIGURE S3 The top 15 adverse event signals at the preferred term level, stratified by gender groups.

SUPPLEMENTARY FIGURE S4 The top 15 adverse event signals at the preferred term level, stratified by reported person groups.

SUPPLEMENTARY TABLE S1 All names used in the search of adverse drug event reports related to linezolid in the FAERS database, including generic names, brand names, and nonstandard names.

SUPPLEMENTARY TABLE S2 Signal strength of ADE reports for linezolid at the System Organ Class (SOC) level in the FAERS database. Positive results that surpass the disproportionality analysis thresholds are highlighted in red. ADE, adverse drug event.

SUPPLEMENTARY TABLE S3 The 263 PT entries that simultaneously satisfied the 4 methods of disproportionality analysis with positive signal values. PT entries are displayed in descending order of case number. Asterisks (*) indicate unexpected signals that are not indicated in the drug label.

SUPPLEMENTARY TABLE S4 Gender-based subgroup analysis of linezolid-related ADEs. a, number of reports of target ADE with linezolid in female; b, number of reports of other ADEs with linezolid in female; c, number of reports of target ADE with linezolid in male; d, number of reports of other ADEs with linezolid in male. Therefore, the ROR here is not a strictly defined ROR in pharmacoepidemiological perspective; we just use this method for signal value calculation of gender-based, linezolid-related signal strength differences.

SUPPLEMENTARY TABLE S5 Weibull distribution test for overall and subgroups of TTO analysis. IQR, interquartile range; Min, minimum; Max: maximum.

SUPPLEMENTARY TABLE S6 A more detailed TTO analysis at the SOC and PT levels. Min, minimum; Max: maximum; IQR, interquartile range; q1, 1/4 quantile; q3, 3/4 quantile; SD, standard deviation; SE, standard error.
