# Supplementary material for: Adverse drug events associated with linezolid administration: a real-world pharmacovigilance study from 2004 to 2023 using the FAERS database
Source: Front Pharmacol. 2024 Feb 16;15:1338902. doi: 10.3389/fphar.2024.1338902 (PMC10904462; doi:10.3389/fphar.2024.1338902)
Supplement: Supplementary file 1 [file Table8.DOCX]

**Overall**

| **Cases**  **n** | **TTO (days)** | | **Weibull distribution** | | | | **Failure type** |
| --- | --- | --- | --- | --- | --- | --- | --- |
|  |  |  | **Scale parameter** | | **Shape parameter** | |  |
|  | **Media (IQR) Min-Max** | | **α 95% CI** | | **β 95% CI** | |  |
| 4362 | 6 (1-15) | 0-2929 | 18.77 | 17.72-19.82 | 0.62 | 0.61-0.64 | Early failure |

**Age**

**<18**

| **Cases**  **n** | **TTO (days)** | | **Weibull distribution** | | | | **Failure type** |
| --- | --- | --- | --- | --- | --- | --- | --- |
|  |  |  | **Scale parameter** | | **Shape parameter** | |  |
|  | **Media (IQR) Min-Max** | | **α 95% CI** | | **β 95% CI** | |  |
| 6  **18-64** | 9.5 (2.3-18.3) | 0-26 | 18.71 | 11.04-26.38 | 2.53 | 0.51-4.55 | Random failure |
| **Cases**  **n** | **TTO (days)** | | **Weibull distribution** | | | | **Failure type** |
|  |  |  | **Scale parameter** | | **Shape parameter** | |  |
|  | **Media (IQR) Min-Max** | | **α 95% CI** | | **β 95% CI** | |  |
| 73 | 3 (0-11) | 0-284 | 17.73 | 8.86-26.60 | 0.57 | 0.46-0.67 | Early failure |
| **Cases**  **>64**  **n** | **TTO (days)** | | **Weibull distribution** | | | | **Failure type** |
|  |  |  | **Scale parameter** | | **Shape parameter** | |  |
|  | **Media (IQR) Min-Max** | | **α 95% CI** | | **β 95% CI** | |  |
| 70 | 4 (1-8) | 0-43 | 8.81 | 6.46-11.15 | 1.06 | 0.86-1.27 | Random failure |

**Gender**

**Male**

| **Cases**  **n** | **TTO (days)** | | **Weibull distribution** | | | | **Failure type** |
| --- | --- | --- | --- | --- | --- | --- | --- |
|  |  |  | **Scale parameter** | | **Shape parameter** | |  |
|  | **Media (IQR) Min-Max** | | **α 95% CI** | | **β 95% CI** | |  |
| 2380 | 6 (2-16) | 0-2929 | 19.84 | 18.32-21.35 | 0.61 | 0.59-0.63 | Early failure |
| **Cases**  **Female**  **n** | **TTO (days)** | | **Weibull distribution** | | | | **Failure type** |
|  |  |  | **Scale parameter** | | **Shape parameter** | |  |
|  | **Media (IQR) Min-Max** | | **α 95% CI** | | **β 95% CI** | |  |
| 1676 | 5 (1-14) | 0-2557 | 17.99 | 16.37-19.61 | 0.64 | 0.61-0.66 | Early failure |

**Weight**

**<80kg**

| **Cases**  **n** | **TTO (days)** | | **Weibull distribution** | | | | **Failure type** |
| --- | --- | --- | --- | --- | --- | --- | --- |
|  |  |  | **Scale parameter** | | **Shape parameter** | |  |
|  | **Media (IQR) Min-Max** | | **α 95% CI** | | **β 95% CI** | |  |
| 1586  **80-100kg** | 6 (1-16) | 0-2929 | 20.69 | 18.64-22.75 | 0.58 | 0.56-0.60 | Early failure |
| **Cases**  **n** | **TTO (days)** | | **Weibull distribution** | | | | **Failure type** |
|  |  |  | **Scale parameter** | | **Shape parameter** | |  |
|  | **Media (IQR) Min-Max** | | **α 95% CI** | | **β 95% CI** | |  |
| 496 | 5 (1-14) | 0-904 | 19.34 | 16.11-22.57 | 0.64 | 0.59-0.68 | Early failure |
| **Cases**  **>100kg**  **n** | **TTO (days)** | | **Weibull distribution** | | | | **Failure type** |
|  |  |  | **Scale parameter** | | **Shape parameter** | |  |
|  | **Media (IQR) Min-Max** | | **α 95% CI** | | **β 95% CI** | |  |
| 240 | 7 (1-17) | 0-1764 | 19.93 | 15.08-24.78 | 0.61 | 0.56-0.67 | Early failure |

**Reported person**

| **Cases**  **Consumer**  **n** | **TTO (days)** | | **Weibull distribution** | | | | **Failure type** |
| --- | --- | --- | --- | --- | --- | --- | --- |
|  |  |  | **Scale parameter** | | **Shape parameter** | |  |
|  | **Media (IQR) Min-Max** | | **α 95% CI** | | **β 95% CI** | |  |
| 514  **Health professional** | 2 (0-7) | 0-1764 | 15.01 | 11.80-18.21 | 0.54 | 0.50-0.58 | Early failure |
| **Cases**  **n** | **TTO (days)** | | **Weibull distribution** | | | | **Failure type** |
|  |  |  | **Scale parameter** | | **Shape parameter** | |  |
|  | **Media (IQR) Min-Max** | | **α 95% CI** | | **β 95% CI** | |  |
| 3641 | 6 (2-16) | 0-2929 | 18.94 | 17.83-20.05 | 0.65 | 0.63-0.66 | Early failure |
