# Supplementary figures and images for: Adverse drug events associated with linezolid administration: a real-world pharmacovigilance study from 2004 to 2023 using the FAERS database
Source: Front Pharmacol. 2024 Feb 16;15:1338902. doi: 10.3389/fphar.2024.1338902 (PMC10904462; doi:10.3389/fphar.2024.1338902)

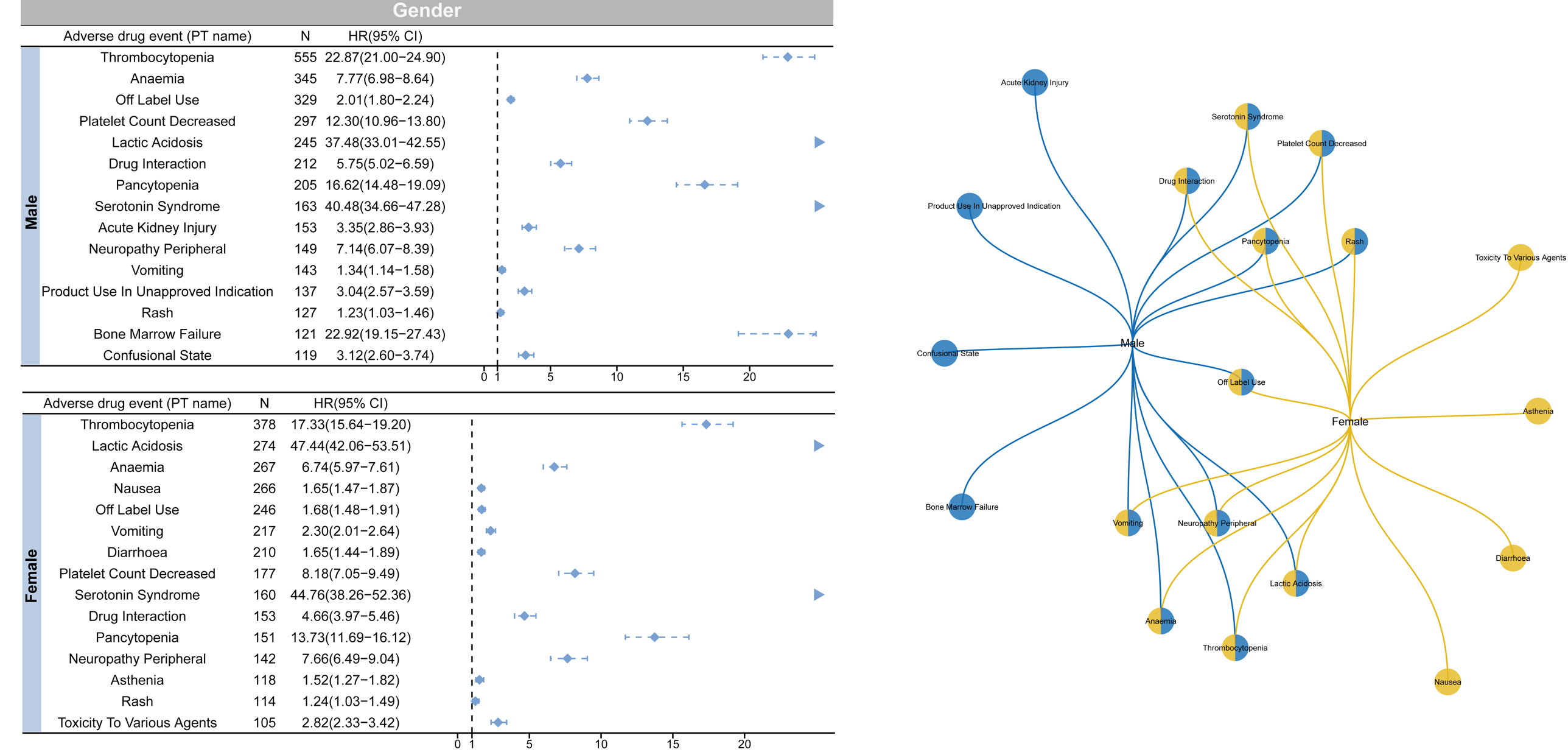

Supplement: Supplementary file 3 [file Image3.TIF]

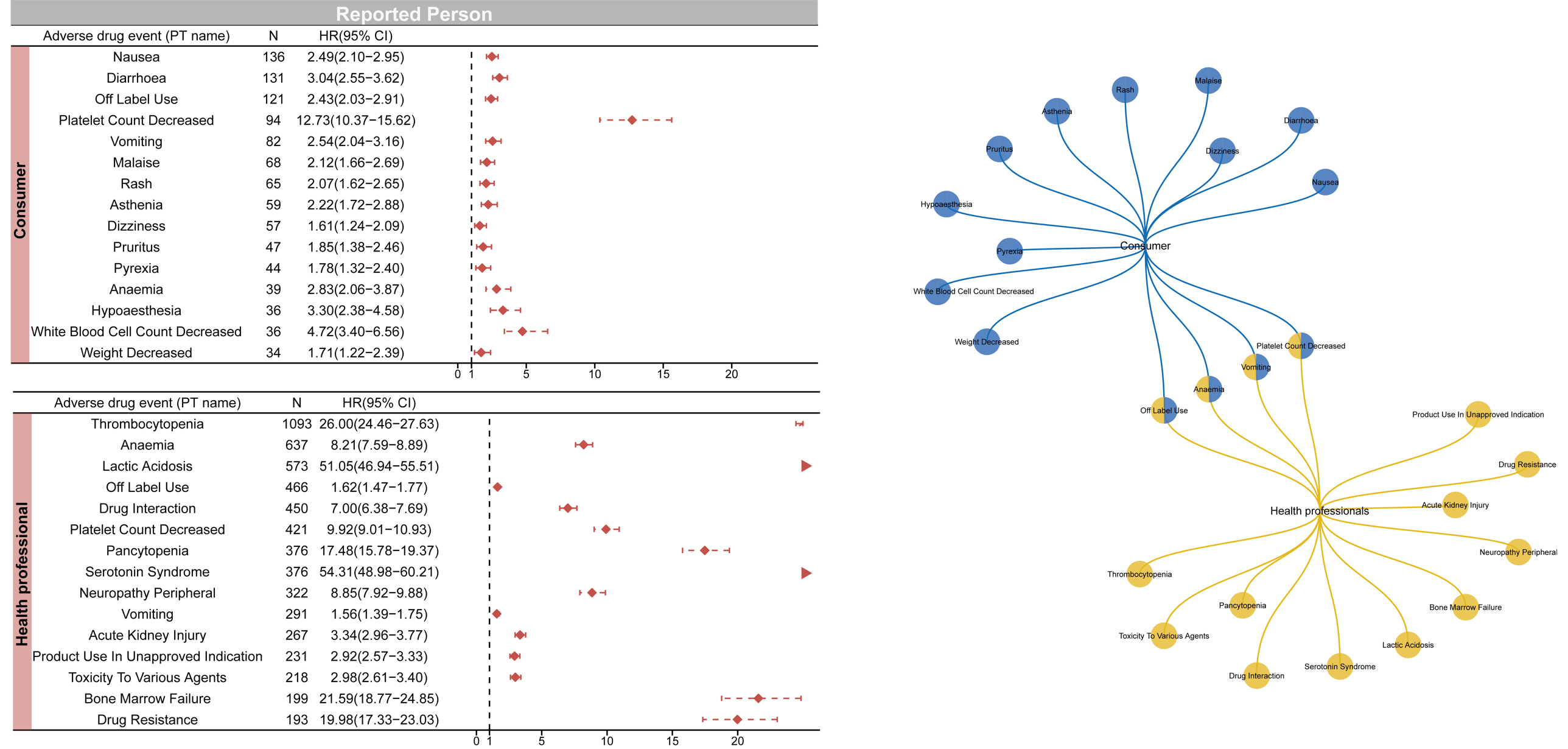

Supplement: Supplementary file 4 [file Image4.TIF]

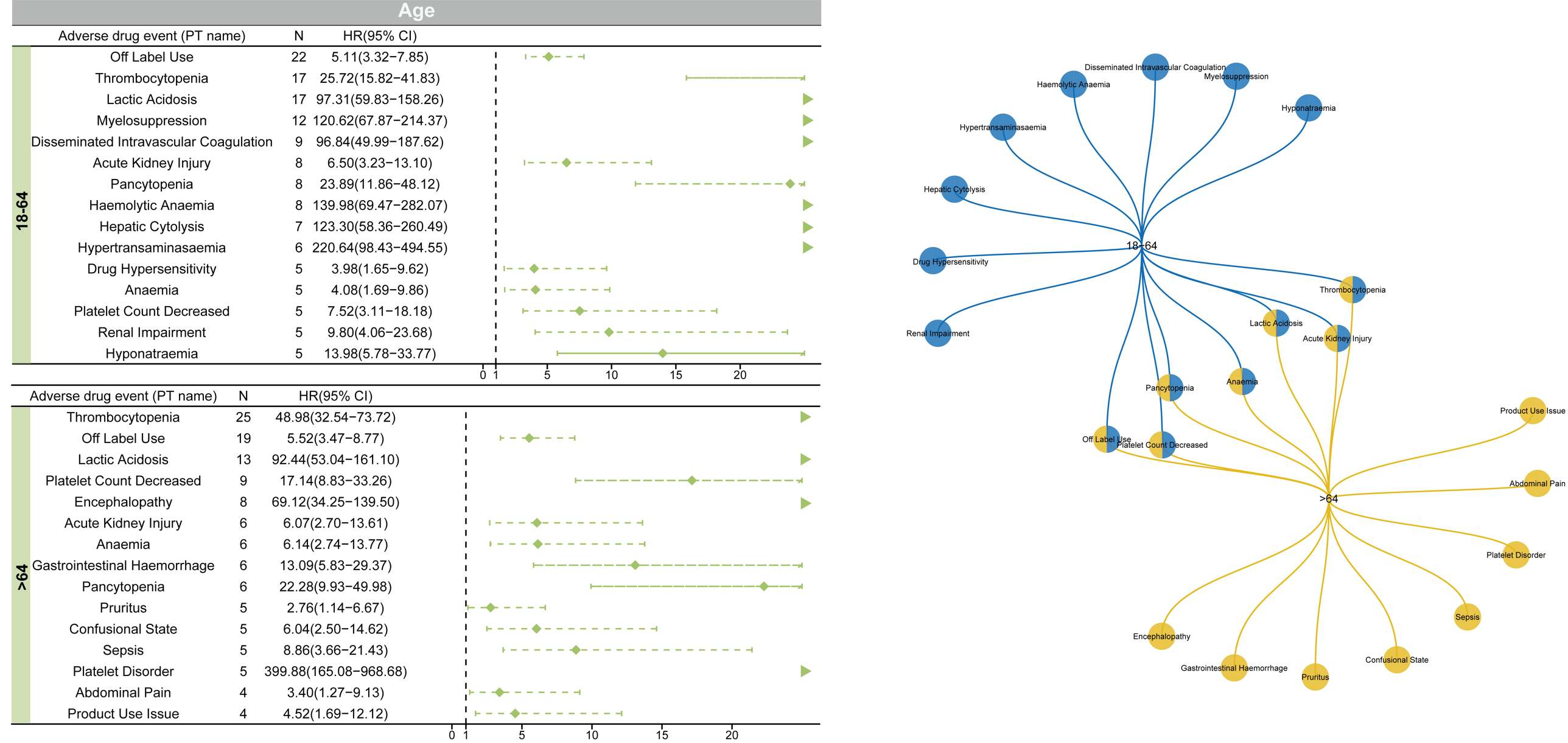

Supplement: Supplementary file 5 [file Image2.TIF]
